# Mpe1 senses the binding of pre-mRNA and controls 3′ end processing by CPF

**DOI:** 10.1016/j.molcel.2022.04.021

**Published:** 2022-07-07

**Authors:** Juan B. Rodríguez-Molina, Francis J. O’Reilly, Holly Fagarasan, Eleanor Sheekey, Sarah Maslen, J. Mark Skehel, Juri Rappsilber, Lori A. Passmore

**Affiliations:** 1 MRC Laboratory of Molecular Biology, Cambridge CB2 0QH, UK; 2 Technische Universität Berlin, Chair of Bioanalytics, 10623 Berlin, Germany; 3 Wellcome Centre for Cell Biology, University of Edinburgh, Edinburgh EH9 3BF, UK

**Keywords:** cryo-EM, poly(A) tail, mRNA, transcription, polyadenylation, endonuclease, polymerase, 3' end processing, transcription termination

## Abstract

Most eukaryotic messenger RNAs (mRNAs) are processed at their 3′ end by the cleavage and polyadenylation specificity factor (CPF/CPSF). CPF mediates the endonucleolytic cleavage of the pre-mRNA and addition of a polyadenosine (poly(A)) tail, which together define the 3′ end of the mature transcript. The activation of CPF is highly regulated to maintain the fidelity of RNA processing. Here, using cryo-EM of yeast CPF, we show that the Mpe1 subunit directly contacts the polyadenylation signal sequence in nascent pre-mRNA. The region of Mpe1 that contacts RNA also promotes the activation of CPF endonuclease activity and controls polyadenylation. The Cft2 subunit of CPF antagonizes the RNA-stabilized configuration of Mpe1. *In vivo*, the depletion or mutation of Mpe1 leads to widespread defects in transcription termination by RNA polymerase II, resulting in transcription interference on neighboring genes. Together, our data suggest that Mpe1 plays a major role in accurate 3′ end processing, activating CPF, and ensuring timely transcription termination.

## Highlights

• The Mpe1 subunit of CPF directly connects pre-mRNA recognition to the 3′ endonuclease
• Mpe1 contacts pre-mRNA directly via a PSR
• The PSR of Mpe1 is required for efficient cleavage and regulated polyadenylation
• Depletion or mutation of Mpe1 in yeast impairs transcription termination

## Graphical abstract

**Figure:**
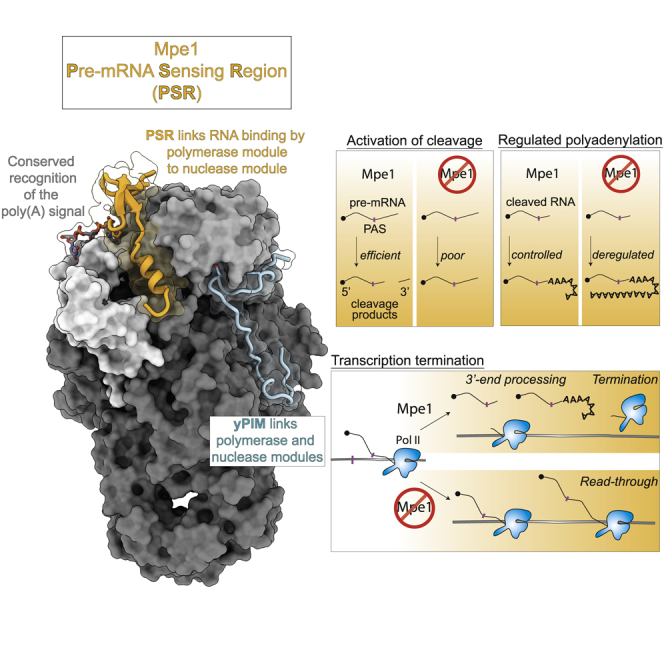

## Teaser

Cleavage and polyadenylation factor (CPF) mediates the 3′ end processing of eukaryotic mRNAs. Rodríguez-Molina et al. show that the Mpe1 subunit contacts the polyadenylation signal in RNA through a pre-mRNA-sensing region (PSR). They show that the PSR promotes pre-mRNA cleavage, controls polyadenylation, and ensures timely transcription termination to safeguard the transcriptome.

## Introduction

Co-transcriptional processing of pre-messenger RNAs (pre-mRNAs), including 5′-capping, splicing, and 3′ end processing, is crucial for their nuclear export, cellular localization, stability, and translation (Hocine et al., 2010; Moore and Proudfoot, 2009). mRNA 3′ end processing involves specific endonucleolytic cleavage and the addition of a polyadenosine (poly(A)) tail onto the new 3′ end by the cleavage and polyadenylation factor (CPF in yeast and CPSF in human) (Kumar et al., 2019; Sun et al., 2020). Endonucleolytic cleavage releases pre-mRNA from the site of transcription and creates an exposed 5′ monophosphate in the RNA polymerase II (RNAPII)-bound nascent RNA. The unprotected 5′ end serves as a substrate for the torpedo exonuclease (Rat1), which degrades the downstream RNA and displaces RNAPII from chromatin, promoting transcription termination (Kim et al., 2004). Thus, controlled cleavage by CPF/CPSF defines the 3′ UTR sequence of the mRNA and is also required for transcription termination.

CPF subunits, and 3′ end processing in general, are highly conserved across all eukaryotes. In the yeast *Saccharomyces cerevisiae*, CPF is assembled into a 14-subunit (∼850 kDa) complex, which is organized into three enzymatically distinct and interconnected modules: the polymerase, nuclease, and phosphatase modules (Casañal et al., 2017). The polymerase and nuclease modules are better characterized than the phosphatase module.

The polymerase module (mammalian polyadenylation specificity factor (mPSF) in humans) serves as the central interaction hub for the 3′ end processing machinery and harbors the poly(A) polymerase, Pap1. Cryo-EM structures of the core polymerase module from yeast and human revealed an assembly of four beta propellers within Cft1 and Pfs2 (CPSF160 and WDR33 in human) (Casañal et al., 2017; Clerici et al., 2017, 2018; Sun et al., 2018). These act as a scaffold for the RNA-binding subunit Yth1 and the Pap1-binding subunit Fip1 (CPSF30 and FIP1 in human) (Casañal et al., 2017).

The nuclease module contains the endonuclease Ysh1, the pseudo-nuclease Cft2, and the multidomain protein Mpe1 (orthologs of human CPSF73, CPSF100, and RBBP6, respectively). A conserved N-terminal ubiquitin-like domain (UBL) in Mpe1 interacts with the nuclease domain of Ysh1 (Hill et al., 2019). A zinc knuckle and RING finger in Mpe1 are thought to interact with nascent pre-mRNAs, possibly just upstream of the cleavage site, and are important for CPF function (Baejen et al., 2014; Lee and Moore, 2014). Functionally, Mpe1 and RBBP6 stimulate cleavage and help select the cleavage site (Di Giammartino et al., 2014; Lee and Moore, 2014; Vo et al., 2001). Mpe1 also stimulates polyadenylation (Lee and Moore, 2014; Vo et al., 2001).

The activation of the CPF endonuclease is highly controlled and requires the coordinated assembly of CPF with two accessory RNA-binding factors, cleavage factors IA and IB (CF IA and CF IB) (Gordon et al., 2011; Gross and Moore, 2001; Hill et al., 2019; Kessler et al., 1997; Kumar et al., 2019). CPF, CF IA, and CF IB each bind to specific sequence elements in pre-mRNAs, and together they activate the 3′ end processing machinery.

The polyadenylation signal (PAS), which is also referred to as the positioning element in yeast, is conserved from yeast to mammals with a consensus sequence of A_1_A_2_U_3_A_4_A_5_A_6_ (Guo and Sherman, 1996; Russo et al., 1993; Tian and Graber, 2012). The structures of mPSF show that CPSF30 zinc finger 2 binds A_1_ and A_2_ of the PAS, and zinc finger 3 binds A_4_ and A_5_ (Clerici et al., 2018; Sun et al., 2018). U_3_ and A_6_ form a Hoogsteen base pair that inserts into a hydrophobic pocket of WDR33 (Clerici et al., 2018; Sun et al., 2018). In yeast, the PAS sequence is more degenerate, but Yth1 zinc fingers 2 and 3 are predicted to recognize the A_1_A_2_ and A_4_A_5_ dinucleotides, similar to PAS recognition in humans. By contrast, the N-terminal loop of WDR33 that binds the U_3_:A_6_ Hoogsteen base pair is not conserved in the yeast counterpart, Pfs2. It remains unclear how PAS recognition results in endonuclease activation.

Here, we present structural, biochemical, and transcriptomic evidence that Mpe1 binds the polymerase module and that, surprisingly, Mpe1 makes direct contact with the PAS RNA. We show that the residues of Mpe1 that contact the polymerase module, RNA, and Ysh1 are required for the efficient activation of cleavage, regulated polyadenylation, and transcription termination. Overall, this suggests that Mpe1 senses RNA binding by CPF and regulates cleavage, polyadenylation, and transcription termination.

## Results

### Mpe1 interacts directly with the polymerase module

We first investigated whether Mpe1 links the nuclease and polymerase modules through a direct interaction with any of the five subunits of the polymerase module. Preliminary experiments using pairwise co-expression in insect cells suggest that Cft1 (but no other polymerase module subunits) copurified with StrepII-tagged Mpe1 (Mpe1-SII) (Figure S1A). We also found that Mpe1 and a purified polymerase module form a complex that could be purified by size exclusion chromatography, although Mpe1 was associated at substoichiometric levels (Figure S1B).

An Mpe1 construct including the zinc knuckle and the downstream linker was previously shown to interact with Cft1 in a yeast-two-hybrid assay (Lee and Moore, 2014). In agreement with this, the removal of the Mpe1 zinc knuckle abolished Mpe1 association with the polymerase module (Figure S1C), and a construct comprising only the Mpe1 zinc knuckle and downstream linker bound but more weakly than wild-type (WT) Mpe1 (Figure S1D). Hydrogen-deuterium exchange followed by mass spectrometry (HDX-MS) showed that several regions of Mpe1 are protected from solvent exchange upon interaction with the polymerase module (Figure S1E). This indicates that Mpe1 may make multiple contacts with the polymerase module or that Mpe1 may rearrange upon binding.

Because Mpe1 had previously been implicated in RNA binding (Baejen et al., 2014; Lee and Moore, 2014), we next tested whether the polymerase module and Mpe1 form a stable complex with RNA. For these experiments, we used the 3′ end of the *CYC1* transcript that is often used as a model substrate for *in vitro* cleavage and polyadenylation assays (Hill et al., 2019). Specifically, we used a 42-nt 5′ FAM-labeled “precleaved *CYC1*” RNA that corresponds to the 5′ product of the cleavage reaction and includes the AAGAA PAS sequence (see Figure 2A). In size exclusion chromatography, the polymerase module, Mpe1, and RNA comigrated (Figure S1B). RNA promoted a more stoichiometric association of Mpe1 with the polymerase module. Interestingly, RNA shifted the polymerase module-Mpe1 complex to a later elution volume, consistent with a potential conformational change. Together, these data suggest that in the presence of RNA, Mpe1 interaction with the polymerase module is stabilized, and the complex may undergo a conformational change.

**Figure 1:**
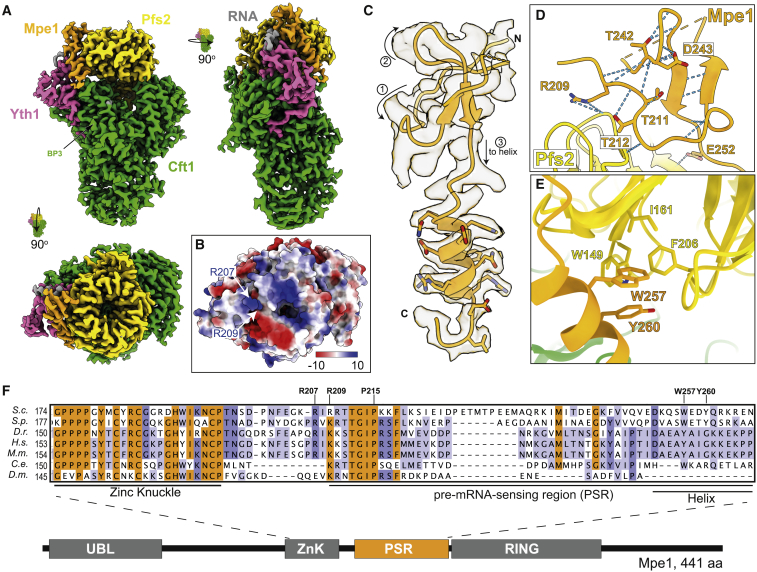
Structure of Mpe1 bound to the polymerase module of CPF (A) Cryo-EM map of the polymerase module in a complex with Mpe1 and RNA. Beta-propeller 3 (BP3) of Cft1 is indicated. (B) Surface representation of polymerase module-Mpe1-RNA (looking down the center of the Pfs2 beta-propeller), colored by electrostatic potential (±10 kT/e). Highlighted residues (R207 and R209) belong to Mpe1. (C) Cartoon representation of residues 207–268 of the Mpe1 pre-mRNA-sensing region (PSR) within a corresponding section of the cryo-EM map. The direction of the polypeptide chain is shown with arrows and numbered 1–3. The N and C termini are labeled. (D) Hydrogen bond network (blue dashed lines) within Mpe1 residues 207–252. Side chains involved in hydrogen bonds are shown in sticks; all other hydrogen bonds are with main-chain atoms. In (C) and (D), orange dashes denote a disordered region that is not visible in the map (residues 224–239). (E) Selected residues of the Mpe1 PSR helix (orange, W257 and Y260) and the hydrophobic pocket of Pfs2 (yellow). (F) Multiple sequence alignment of the zinc knuckle and PSR of Mpe1 orthologs. Residues highlighted in orange are conserved; those in purple are partially conserved. A domain diagram of Mpe1 is shown below. *S.c., Saccharomyces cerevisiae*; *S.p*., *Schizosaccharomyces pombe*; *D.r*., *Danio rerio*; *H.s*., *Homo sapiens*; *M.m.*, *Mus musculus*; *C.e*., *Caenorhabditis elegans*; and *D.m*., *Drosophila melanogaster*. See also Figures S1 and S2 and Video S1.

Next, we analyzed the polymerase module-Mpe1-RNA complex by single-particle electron cryomicroscopy (cryo-EM) (Table 1; Figures S1F and S1G). Compared with the previous structure of the yeast polymerase module (Casañal et al., 2017), the sample we used here additionally contained Pap1, Mpe1, and the precleaved *CYC1* RNA. We obtained a map of the complex at an overall resolution of 2.7 Å (Figures 1A and S1H–S1K). In this map, we identified an additional density, which is not present in the previous maps of the yeast polymerase module or mPSF (Casañal et al., 2017; Zhang et al., 2020), that extends from the top of Pfs2 toward Yth1 and Cft1 (Figure 1A, orange). There is also a poorly ordered density on top of the Pfs2 beta-propeller and in front of the C-terminal helical domain of Cft1 (Figure S2A).

**Table 1:** Cryo-EM data collection, model refinement, and validation statistics

	Polymerase module-Mpe1-RNA (PDB: 7ZGP, EMDB: EMD-14710)	Polymerase module-Cft2(S) (PDB: 7ZGQ, EMDB: EMD-14711)	Polymerase module-Mpe1-yPIM-RNA (PDB: 7ZGR, EMDB: EMD-14712)
**Data collection and processing**			

Magnification	105,000 ×	105,000 ×	105,000 ×
Voltage (kEV)	300	300	300
Electron exposure (e^−^/Å^2^)	40	37	40
Defocus range (μm)	−0.5 to −3.1	−0.5 to −3.1	−0.5 to −3.1
Pixel size (Å)	0.83 (eBIC)	0.86 (LMB)	0.86 (LMB)
Symmetry imposed	C1	C1	C1
Initial particle images (no.)	6,460,073	1,946,027	13,905,256
Final particle images (no.)	131,152	141,584	846,349
Map resolution (Å)	2.66	2.79	2.61
FSC threshold	0.143	0.143	0.143
Map resolution range (Å)	2.66 to >10	2.79 to >10	2.61 to >10

**Refinement**			

Initial model used	*de novo* modeling and polymerase module (PDB: 6eoj)	mPSF-PIM (PDB: 6urg) and polymerase module (PDB: 6eoj)	polymerase module-Mpe1-RNA and polymerase module-Cft2(S)
Model resolution (Å)	–	–	–
FSC threshold	0.143	0.143	0.143
Model resolution range (Å)	–	–	–
Map sharpening *B* factor (Å^2^)	−20	−30	−40

**Model composition**			

Non-hydrogen atoms	14,063	13,704	14,505
Protein residues	1,767	1,749	1,819
Nucleotides	4	0	4
Ligands	ZN:2	ZN:2	ZN:2

***B* factors (Å**^**2**^**)**			

Protein	not estimated	not estimated	not estimated
Ligand			

**RMS deviations**			

Bond lengths (Å)	0.003	0.003	0.003
Bond angles (°)	0.518	0.539	0.537

**Validation**			

MolProbity score	1.97	2.39	1.93
Clashscore	9.55	11.24	8.12
Poor rotamers (%)	1.15	3.25	1.30

**Ramachandran plot**			

Favored (%)	93.63	93.27	94.11
Allowed (%)	6.25	6.73	5.72
Disallowed (%)	0.11	0.0	0.17

Given the resolution of the map, we could *de novo* build an atomic model into a part of the additional density, including a region of Mpe1 downstream of the zinc knuckle (residues 207–222 and 240–268) (Video S1). This region of Mpe1 primarily contacts Pfs2 but also comes in close proximity to Cft1 and Yth1. Residues 223–239 were not resolved in our structure. We identified the remaining well-ordered density as three nucleotides of the PAS of the *CYC1* RNA. We will refer to the region of Mpe1 that is ordered in our maps as the pre-mRNA-sensing region (PSR). Densities for Fip1 and Pap1 were not identified in the map.

**Figure 2:**
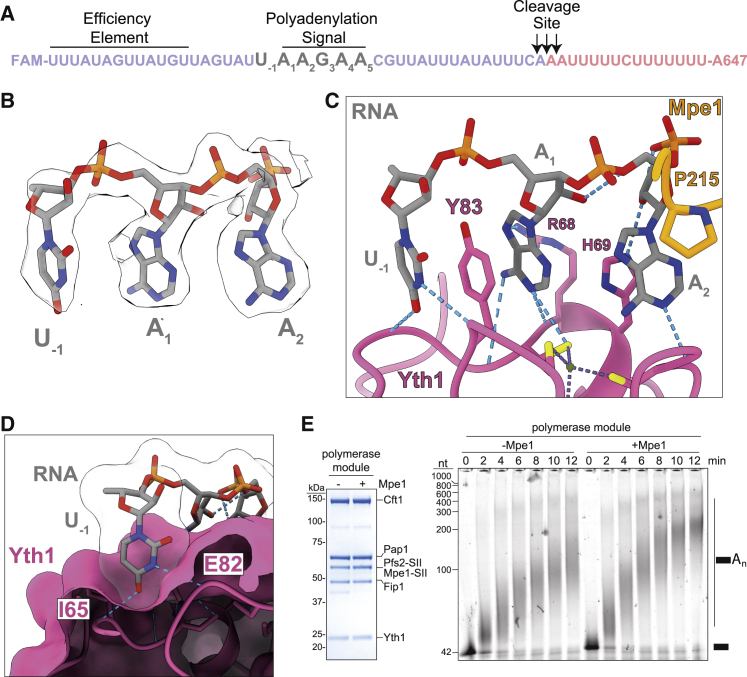
Mpe1 contacts the polyadenylation signal (PAS) in RNA and stimulates polyadenylation (A) Sequence of the *CYC1* RNA substrate. The full sequence is “uncleaved” *CYC1*. (B) Cryo-EM map (transparent surface) and model (sticks) of PAS RNA in the polymerase module-Mpe1-RNA map. (C) Contacts between the PAS of *CYC1* RNA (gray) and Yth1 (pink) and Mpe1 (orange). P215 of Mpe1 contacts A_2_ via a CH-π interaction. Blue dashed lines show hydrogen bonds. (D) The U_−1_ nucleotide (sticks and transparent surface) sits in an open pocket on Yth1 (magenta surface) and makes hydrogen bonds to the main chain of I65 and E82. (E) Polyadenylation activity of polymerase module without or with Mpe1. Left, SDS-PAGE of purified complexes. Right, polyadenylation reactions using a 5′ FAM-labeled precleaved *CYC1* RNA substrate (shown schematically with a black rectangle), analyzed by urea-PAGE. CF IA and CF IB were not included in these reactions. See also Figure S2 and Video S1.

Video S1. Model of polymerase module-Mpe1-yPIM-RNA complex, related to Figures 1 and 2

### Mpe1 contacts Pfs2 and Cft1

Two arginine residues at the N terminus of the Mpe1 PSR (R207 and R209) are positioned next to a positively charged patch on the top surface of Pfs2 (Figure 1B), which was previously predicted to participate in RNA binding (Casañal et al., 2017). Thus, Mpe1 may also contribute to this putative RNA-binding site. Mpe1 residues 207–252 form a small, compact fold that packs against the Pfs2 beta-propeller. This fold is held together by a network of hydrogen bonds and a hydrophobic core (Figures 1C and 1D). Mpe1 then continues as a helix (residues 253–268) that makes additional contacts with the side of the Pfs2 beta-propeller. Two aromatic residues from this helix (W257 and Y260) insert into a hydrophobic pocket of Pfs2 that is lined by W149, I161, and F206 (Figure 1E). Mpe1 is not visible after residue Q268, which is positioned near beta-propeller 3 (BP3) of Cft1. Curiously, although Mpe1 and Cft1 interact directly in pull-downs, there is little direct contact between them in the models. Together, these data reveal an unexpected architecture by which Mpe1 interacts with the polymerase module of CPF.

We aligned the sequences of Mpe1 orthologs from diverse eukaryotic species and found that PSR residues 209–218 are highly conserved (Figure 1F). The loop that is not resolved in our map (residues 223–239) and the helix are not well conserved except for W257, which is conserved as an aromatic residue.

We also compared our model of yeast polymerase module-Mpe1-RNA with the structure of human mPSF. This showed that the Mpe1 PSR helix overlaps with a loop of CPSF30 (residues 22–34). Specifically, F30 of CPSF30 inserts into the hydrophobic pocket of WDR33 that binds Mpe1 W257 in yeast (Figure S2B). F30 of CPSF30 is conserved only among metazoans, but the residues that line the hydrophobic pocket in Pfs2 are mostly conserved in WDR33 (Figures S2C and S2D; Clerici et al., 2018). Thus, although some aspects of Mpe1 interaction with Pfs2 are conserved, the Mpe1 binding pocket is instead occupied by CPSF30 in humans.

### Mpe1 senses RNA binding to the polymerase module

The density near the zinc finger 2 of Yth1 corresponds to the nucleotides A_1_ and A_2_ of the PAS of *CYC1* (A_1_A_2_G_3_A_4_A_5_), as well as one additional upstream nucleotide (U_−1_) (Figures 2A and 2B). There is a weak density for a fourth nucleotide (G_3_). The first two nucleotides in the structure are arranged in a U_−1_-Yth1 Y83-A_1_ stack (Figure 2C). R68 of Yth1 contacts the opposite face of the A_1_ base. The A_2_ base is stabilized by a π-π interaction with the H69 of Yth1. Sequence recognition is mediated by hydrogen bonds to the N1 amino groups of A_1_ and A_2_ and to the N6 amino group of A_1_. This overall mechanism of binding to the A-dinucleotide by yeast Yth1 is very similar to its human counterpart, suggesting that the recognition of the 5′ end of the PAS by zinc finger 2 is conserved (Figures 2C and S2E).

Surprisingly, in addition to the interactions between RNA and Yth1, A_2_ is contacted directly by P215 of Mpe1 through a CH-π interaction (Levitt and Perutz, 1988; Figure 2C). P215 and the surrounding residues are conserved in Mpe1 homologs (Figure 1F), suggesting that this interaction may also be conserved across eukaryotes. Interestingly, Mpe1 binding to the polymerase module is stabilized by RNA, but the RNA-binding affinity of the polymerase module is not substantially affected by Mpe1 (Figures S2F–S2H). We hypothesize that the Mpe1 PSR binds to the polymerase module only after the PAS RNA has been specifically recognized by Yth1. Therefore, Mpe1 may “sense” RNA binding by the polymerase module.

The −1 nucleotide is not visible in previous mPSF-RNA structures. The base of U_−1_ sits in an open pocket on the surface of Yth1 and forms hydrogen bonds with the main-chain amide of I65 (to U_−1_ O4) and with the main-chain carbonyl of E82 (to U_−1_ N3) (Figure 2D). This pocket may contribute to the RNA-binding affinity of Yth1. Zinc finger 3 is expected to recognize the A_3_A_4_ dinucleotide, but it is not visible here, possibly because it is flexible.

We tested the effect of Mpe1 on the *in vitro* polyadenylation activity of the polymerase module with the 5′ FAM-labeled precleaved *CYC1* RNA as a substrate. These data showed that Mpe1 promotes a modest but reproducible increase in the rate of polyadenylation (Figure 2E). The accessory factors CF IA and CF IB interact with the polymerase module and, in addition to activating cleavage, also increase the activity and processivity of polyadenylation (Casañal et al., 2017). In our assays, CF IA and CF IB mask the stimulatory effect of Mpe1 on polyadenylation (Figure S2I). Therefore, Mpe1 and CFs may stimulate polyadenylation activity similarly by providing additional RNA-binding sites and/or correctly positioning RNA on the polymerase module.

Overall, our structure reveals that the recognition of the first two nucleotides of the PAS by the zinc finger 2 of Yth1 is highly conserved across eukaryotes, but surprisingly, the PAS is also contacted by Mpe1.

### Cft2 antagonizes Mpe1 binding to polymerase module

**Figure 3:**
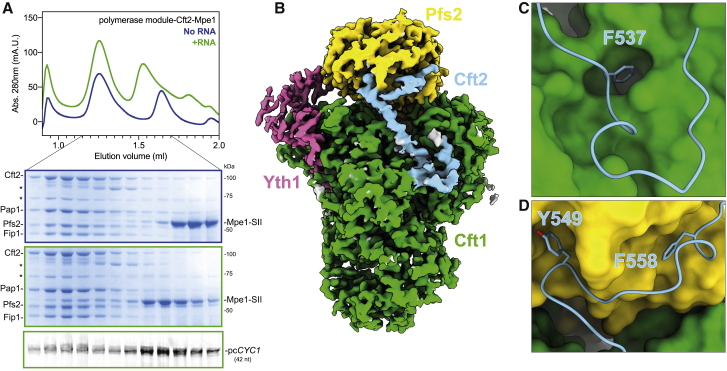
Cft2 antagonizes Mpe1 binding to polymerase module (A) Size exclusion chromatography with polymerase module, Cft2, and Mpe1, with (green) or without (blue) precleaved *CYC1* RNA (pc*CYC1*). Top, chromatogram; middle two panels, Coomassie-stained SDS-PAGE of indicated fractions; and bottom, urea-PAGE of fluorescently labeled RNA from the indicated fractions. The gels are outlined in colors corresponding to the chromatograms. ^∗^ denotes degradation products of Cft2. (B) Cryo-EM map of the polymerase module in complex with Cft2(S). The yeast polymerase module interacting motif (yPIM) of Cft2 is colored in blue. The rest of Cft2, Mpe1, precleaved *CYC1* RNA, Fip1, and Pap1 are not visible in the map. (C and D) The yPIM of Cft2 (blue, cartoon and stick representation) inserts a conserved F537 residue into a hydrophobic pocket in Cft1 (green, surface representation) (C) and conserved Y549 and F558 residues into a hydrophobic pocket of Pfs2 (yellow, surface representation) (D). See also Figure S3

In human CPSF, the polymerase and nuclease modules are tethered together via a conserved peptide motif (mPSF interaction motif, or PIM) in CPSF100 that interacts with a surface groove on CPSF160 (Zhang et al., 2020). To further understand how the yeast nuclease and polymerase modules assemble, we added Cft2 to the polymerase module-Mpe1 complex. In size exclusion chromatography, Cft2 comigrated with the polymerase module, but, surprisingly, this reduced Mpe1 binding (compare Figures 3A and S3A, blue, with Figure S1B). Mpe1 incorporation was recovered by including the precleaved *CYC1* RNA (compare Figures 3A and S3A, green, with Figure S1B). This suggests that there may be contacts among Cft2, Mpe1, and RNA that regulate their binding on the polymerase module.

The Mpe1 UBL domain interacts directly with the endonuclease subunit Ysh1, which in turn binds the C-terminal domain of Cft2 (Dominski et al., 2005; Hill et al., 2019). Thus, Ysh1 stabilizes Mpe1 association with the nuclease module via a mechanism that does not involve RNA. In agreement with this, Mpe1 copurified with a complex of the polymerase module and the nuclease module subunits Ysh1 and Cft2, even in the absence of RNA (Figure S3B).

To determine the architecture of the polymerase module-Cft2-Mpe1-RNA complex, we carried out single-particle cryo-EM. We used a truncated version of Cft2 (Cft2(S), residues 1–720), which is missing the disordered C-terminal region but still interacts with CPF (Figure S3C; Kyburz et al., 2003). We obtained a map of this complex at an overall resolution of 2.8 Å (Figures 3B and S3D–S3G). This map contained density for the polymerase module and a short region of Cft2 but not for Mpe1, the precleaved *CYC1* RNA, Fip1, or Pap1 despite their presence in the cryo-EM specimen.

We built an atomic model of a conserved region of Cft2 that interacts with the polymerase module and is homologous to the human CPSF100 PIM (Zhang et al., 2020). We thus refer to this region of Cft2 (residues 525–562) as the yeast polymerase module interacting motif (yPIM). The yPIM adopts an arrangement on the polymerase module that is highly similar to its human counterpart on the mPSF (Figures S3H–S3J). Conserved aromatic residues in the yPIM (F537, Y549, and F558) stabilize its interaction with the polymerase module by inserting into the hydrophobic pockets in Cft1 and Pfs2 (Figures 3C and 3D). Mutation of these conserved residues strongly impairs the Cft2 interaction with the polymerase module (Figure S3K).

**Figure 4:**
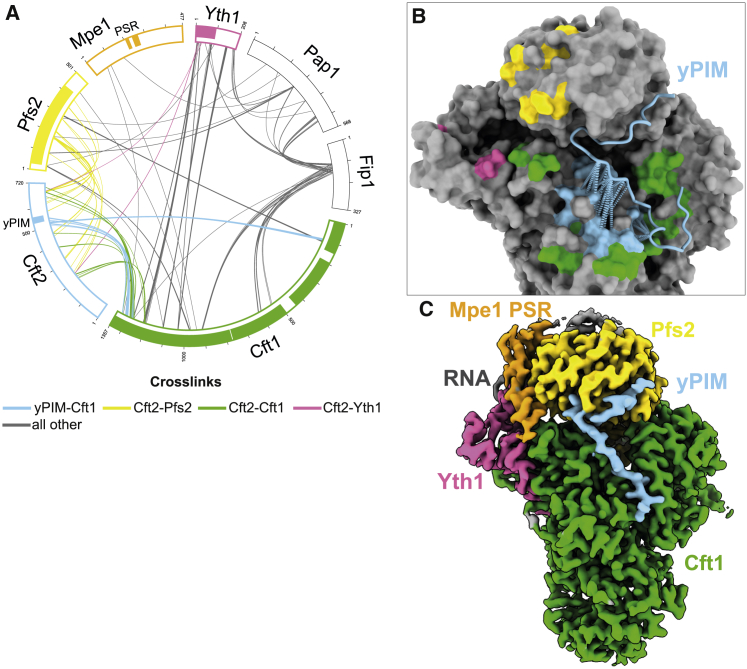
The Cft2 yPIM and Mpe1 PSR can simultaneously bind polymerase module (A) Circular view of the crosslinking mass spectrometry analysis of a polymerase module-Cft2(S)-Mpe1-RNA complex. Each line represents a crosslink. Cft2(S)-polymerase-module crosslinks are in color. Regions that are visible in the cryo-EM structures reported here are indicated with colored boxes around the edge of the circle. (B) Surface representation of the polymerase module-Cft2(S) structure (gray) highlighting regions where Cft2(S) crosslinks to Pfs2 (yellow), Yth1 (pink), and Cft1 (green). Crosslinks between the yPIM and Cft1 are shown as pseudobonds (light blue dotted lines) and light blue surfaces on Cft1. (C) Cryo-EM map of polymerase module-Mpe1-yPIM-RNA complex. The sample for this complex contains polymerase module, Mpe1, a yPIM peptide from Cft2, and the precleaved *CYC1* RNA. See also Figure S4 and supplemental information.

We performed photo-crosslinking using sulfo-NHS-diazirine (sulfo-SDA, sulfosuccinimidyl 4,4′-azipentanoate) followed by mass spectrometry analysis (supplemental information) and did not observe any crosslinks between Cft2 and Mpe1. A cluster of crosslinks confirms that the yPIM binds Cft1 in the groove between beta-propellers 1 and 3 near the Cft1 helical bundle (Figures 4A and 4B). Interestingly, regions of Cft2 both upstream and downstream of the yPIM crosslink in the vicinity of the Mpe1 PSR binding site on Pfs2 (Figures 4A and 4B, yellow). Since we did not observe any Cft2 density in this region of the cryo-EM map, Cft2 may be in close proximity to this part of Pfs2 without forming specific contacts. This suggests that Cft2 may sterically clash with the Mpe1 PSR binding to the polymerase module, even though their binding sites do not directly overlap.

We next determined the impact of an isolated yPIM peptide on complex formation. Substoichiometric amounts of Mpe1 comigrate with the polymerase module in a complex with a synthetic yPIM peptide on size exclusion chromatography (Figure S4A, top gel). Including the precleaved *CYC1* RNA stabilizes Mpe1 on the complex (Figure S4A, middle and bottom gels). In agreement with this, we were able to obtain a cryo-EM map of this complex at a resolution of 2.6 Å that shows that both the Mpe1 PSR and yPIM can bind to the polymerase module simultaneously in the presence of RNA (Figures 4C and S4B–S4E).

Together, these data suggest that the position of Cft2 (but not the yPIM alone) on the polymerase module may sterically hinder Mpe1 PSR binding in the absence of RNA.

### Mpe1 activates CPF cleavage and polyadenylation activity

**Figure 5:**
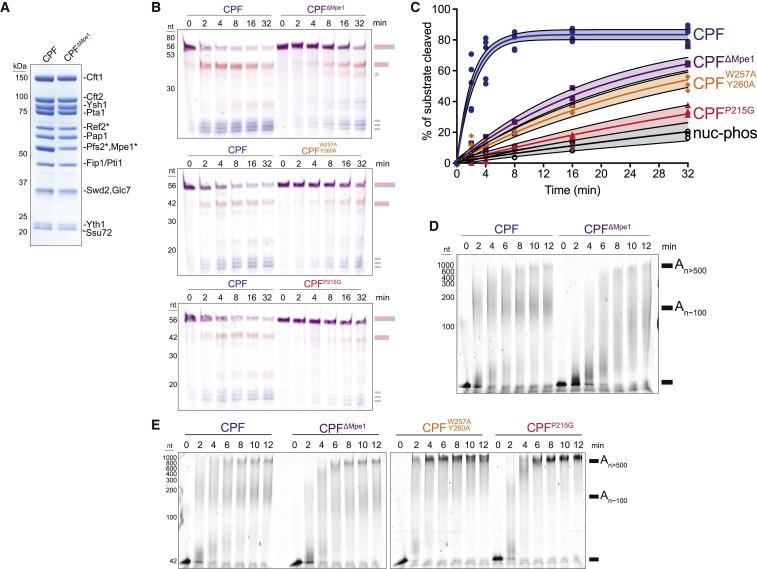
Mpe1 is a regulator of CPF cleavage and polyadenylation (A) SDS-PAGE of CPF with and without Mpe1. Asterisks (^∗^) denote SII-tagged subunits. (B) Representative urea-PAGE of dual-color *in vitro* cleavage assays using an uncleaved *CYC1* RNA substrate (5′ FAM [red] and 3′ Alexa647 [blue] labels) and CPF, CPF^Δ^^M^^pe1^, CPF^W257A/Y260A^, or CPF^P215G^. Cartoons of the substrate and expected RNA products are shown at the right. The asterisk indicates inaccurate cleavage products. (C) Quantitation of cleavage assays (as % of substrate cleaved) using CPF, CPF^Δ^^M^^pe1^, or CPF with mutant Mpe1. For each complex, the fit of the data is shown as a solid line, and the shading represents the 95% confidence interval of the fit. Values for individual replicates are n = 5 for CPF, and n = 3 for all others. “nuc-phos” is the CPF lacking the polymerase module. R^2^ = 0.93–0.97. (D) Urea-PAGE of *in vitro* polyadenylation assay using a 5′ FAM-labeled precleaved *CYC1* RNA substrate. Reactions were carried out with CPF or CPF^Δ^^M^^pe1^, using 100 nM CF IA and IB. (E) Similar to (D) except that CPF with mutant Mpe1 (CPF^W257A/Y260A^ and CPF^P215G^) were included, and reactions were carried out using 450 nM CF IA and IB. See also Figure S5

To determine the functional role of Mpe1 in mRNA 3′ end processing, we purified a fully recombinant 14-subunit CPF with and without Mpe1 (Kumar et al., 2021; Figures 5A and S5A). We performed *in vitro* cleavage assays with CPF, CF IA, and CF IB using a dual-labeled fluorescent “uncleaved” *CYC1* RNA substrate that includes the cleavage site (Figure 2A; Hill et al., 2019). We monitored the cleavage reaction by resolving both the 5′ (FAM-labeled) and 3′ (Alexa647-labeled) cleavage products on a denaturing polyacrylamide gel. Cleavage assays showed that CPF^ΔMpe1^ is ∼10 times slower than full CPF (CPF t_50_ = 1.5 ± 0.27 min, CPF^ΔMpe1^ t_50_ = 14.9 ± 2.5 min; t_50_ is the time needed to cleave half of the maximum RNA cleaved by CPF. The maximum amount of RNA cleaved by CPF is 83.4% of the initial substrate) (Figures 5B and 5C). This suggests that Mpe1 is a major activator of CPF cleavage activity. Interestingly, selection of the cleavage site is less accurate with CPF^Δ^^M^^pe1^ (Figure 5B, asterisk), consistent with a role for Mpe1 in positioning or activating the endonuclease. A CPF complex lacking the polymerase module did not show any cleavage activity (Figures S5B–S5D), confirming the essential role of the polymerase module in 3′ end processing.

To test whether Mpe1 also stimulates polyadenylation activity in the context of full CPF, CF IA, and CF IB, we performed *in vitro* polyadenylation assays using the 5′ FAM-labeled precleaved *CYC1* substrate. Compared with the full complex, CPF lacking Mpe1 shows slower polyadenylation activity and appears to be more distributive (Figure 5D). This suggests that Mpe1 plays an important role in promoting efficient polyadenylation. Thus, Mpe1 serves to both activate cleavage and control polyadenylation by CPF.

### Mpe1 interaction with Ysh1 is important for promoting cleavage and polyadenylation

To test the importance of the interaction between the Mpe1 UBL domain and Ysh1 (Hill et al., 2019), we purified a variant of Mpe1 containing four point mutations in conserved residues at the UBL-Ysh1 interface (F9A, D45K, R76E, and P78G; Mpe1^FDRP^ henceforth). First, to test whether Mpe1^FDRP^ is constitutively incorporated into CPF, we performed size exclusion chromatography at 150 mM KCl. The Mpe1^FDRP^ variant did not assemble into CPF (Figure S5E), suggesting that the Mpe1-Ysh1 interaction is required.

Next, to test the weaker Mpe1-polymerase module interaction, we performed size exclusion chromatography in lower salt (50 mM) and found that Mpe1^FDRP^ co-eluted with the polymerase module in size exclusion chromatography (Figure S5F), comparable to its WT counterpart (Figure S1B). Given that Cft2 antagonizes the interaction between the Mpe1 PSR and the polymerase module (Figure S3A), it is possible that Cft2 also prevents Mpe1 from incorporating into CPF when the Mpe1-Ysh1 interaction is disrupted. Additional experiments are required to determine this conclusively.

Next, we reasoned that although Mpe1^FDRP^ did not form a stable complex with CPF, its interaction with polymerase module might be sufficient to activate cleavage. To test this possibility, we used the purified CPF^Δ^^M^^pe1^ complex in the dual-color *in vitro* cleavage assay and added 4× molar excess of Mpe1 in *trans*. The addition of WT Mpe1 activated the CPF cleavage activity but the addition of Mpe1^FDRP^ did not (Figure S5G).

We also tested whether the addition of WT or Mpe1^FDRP^ could stimulate the polyadenylation activity of CPF. We found that WT Mpe1 restored CPF polyadenylation activity, but Mpe1^FDRP^ could only partially rescue it (Figure S5H). Together, these data are consistent with a role for the Mpe1 UBL in stably tethering Mpe1 to CPF to promote both cleavage and regulated polyadenylation.

### Mpe1 PSR is required for cleavage and regulated polyadenylation

To test the functional relevance of the Mpe1 PSR, we generated mutants of Mpe1 that would disrupt its interaction with Pfs2 (W257A/Y260A) or its direct contact with the A_2_ of the PAS RNA (P215G). Both mutants could be incorporated into recombinant CPF (Figure S5I). These mutants also bind to the polymerase module, but unlike WT Mpe1, RNA did not shift the complex to a later elution volume on a size exclusion column (Figure S5J), suggesting that RNA does not induce the same conformational change.

We next tested the cleavage and polyadenylation activities of CPF complexes carrying each of the Mpe1 PSR mutants. Both mutant complexes show dramatically reduced endonuclease activity compared with WT CPF or CPF^Δ^^M^^pe1^ (CPF^W257A/Y260A^ t_50_ = 21.1 ± 3.9 min and CPF^P215G^ t_50_ = 46.3 ± 12.1 min) (Figures 5B and 5C). Surprisingly, in polyadenylation reactions, both PSR mutant complexes show aberrant hyperpolyadenylation compared with WT or CPF^Δ^^M^^pe1^ complexes (Figure 5E). Thus, the same residues that mediate PSR binding to RNA and Pfs2 are also required for activating cleavage and regulating polyadenylation. Interestingly, Mpe1 PSR mutants cause more severe defects in both cleavage and polyadenylation than the complete loss of Mpe1, suggesting that the presence of a defective Mpe1 might prevent the other subunits of CPF, CF IA, or CF IB from compensating for the lack of Mpe1.

### Mpe1 is required for timely transcription termination

The latent cleavage activity of CPF^Δ^^M^^pe1^ raised the possibility that CPF may still cleave nascent RNA and commit RNAPII to termination in cells, even in the absence of Mpe1. To address this possibility, we investigated the consequence of acute Mpe1 depletion on the yeast transcriptome. We inserted a mini-auxin-induced degron (mAID) (Tanaka et al., 2015) at the C-terminal end of the endogenous *MPE1* locus (Mpe1-mAID). Mpe1 is depleted upon the addition of auxin, resulting in a dose-dependent growth arrest (Figures S6A and S6B). To circumvent buffering mechanisms that could potentially mask the immediate impact of Mpe1 depletion on the transcriptome (Haimovich et al., 2013; Rodríguez-Molina et al., 2016; Sun et al., 2013), we labeled nascent transcripts using 4-thiouracil, which enables their biotinylation and isolation (Sun et al., 2012; Figure S6C).

We analyzed untreated or auxin-treated total and nascent RNA fractions from the WT and Mpe1-mAID cells. Using RT-qPCR, we found that Mpe1 depletion primarily impacted mRNA genes with very little impact on small nucleolar RNA genes (Figure S6D). This is consistent with a gene-class-specific function of CPF in the 3′ end processing of mRNA genes (Lidschreiber et al., 2018). Sequencing revealed that Mpe1 depletion caused a significant change in the nascent RNA levels of 2,623 genes in Mpe1-mAID cells (1,459 increased and 1,164 decreased, log_2_-fold change >0, FDR-adjusted p value < 0.05) but had no significant impact on the transcripts in WT cells (Figure S6E). The impact on the nascent RNA fraction was stronger than that on total RNA (Figure S6E). We, therefore, focused subsequent analyses on the nascent fraction, which would more faithfully reflect the immediate impact of Mpe1 depletion.

**Figure 6:**
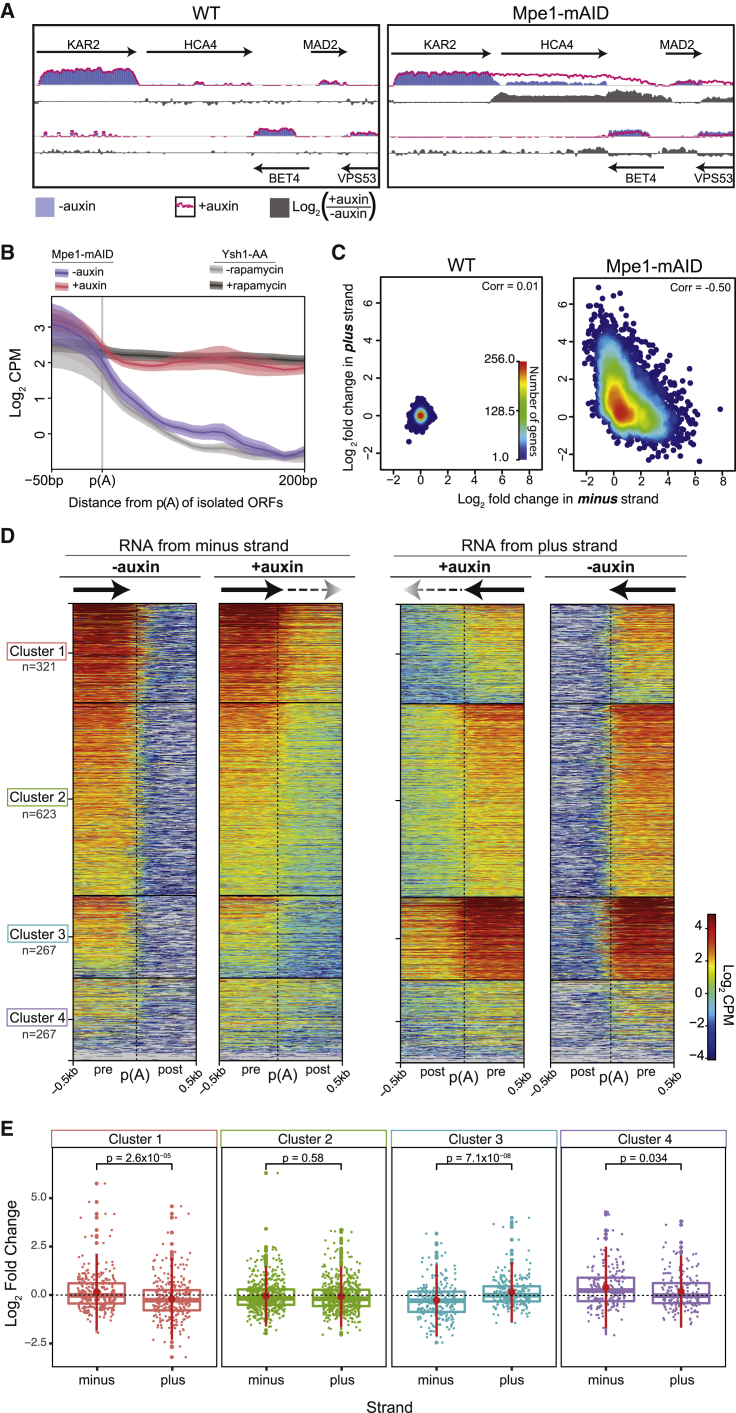
Mpe1 is globally required for timely transcription termination (A) Representative genomic snapshots of strand-specific nascent RNA-seq from WT (left) or Mpe1-mAID (right) yeast, either untreated (blue bars) or treated with auxin (magenta trace). The log_2_-fold change in nascent RNA upon the addition of auxin is in gray. Arrows represent protein-coding genes. (B) Metagene plots of nascent RNA at the polyadenylation site (poly(A)) from the Mpe1-mAID cells treated with auxin (magenta) or untreated (blue). Nascent RNA from Ysh1 anchor away cells (Ysh1-AA), where Ysh1 was depleted (+rapamycin, dark gray) or not depleted from the nucleus (−rapamycin, light gray) is also shown. Ysh1 depletion data were obtained in a previous study (Baejen et al., 2017) and re-analyzed here. Selected genes are ≥200 bp from neighboring ORFs (n = 931 genes). Center line of each curve represents average signal; shaded area is 95% confidence interval. (C) Density scatter plot of changes in nascent RNA synthesis in WT or Mpe1-mAID cells upon the addition of auxin. Values correspond to strand-specific log_2_-fold change per gene and corresponding position on the opposite strand. (D) k-means clustering of strand-specific nascent RNA before and after auxin treatment in Mpe1-mAID cells. Data are shown for a 1-kb window centered around the polyadenylation site (poly(A)) of 1,478 convergent gene pairs. Transcription directionality is indicated with arrows. The number of genes in each cluster (n) is indicated. CPM, counts per million. (E) Log_2_-fold change in nascent RNA upon Mpe1 depletion for the genes in each of the clusters in (D) on the minus and plus strands. Dots represent the log_2_-fold change for each gene. Large dots represent outliers within each distribution. p values are from pairwise Student’s t test. Middle horizontal line in each boxplot represents the median, and the boxes show the interquartile range. Number of genes (n) for each cluster is shown in (D). See also Figures S6 and S7.

Mpe1 depletion led to a widespread increase in the signal downstream of the polyadenylation site of mRNA genes (Figures 6A and 6B, red). The nascent RNA downstream of the cleavage site is normally rapidly degraded after CPF-mediated cleavage of the pre-mRNA. Thus, this is consistent with defects in both 3′ end processing and transcription termination and is indicative of RNAPII readthrough beyond the normal 3′ end of the transcript. The termination defect upon the depletion of Mpe1 was similar to the previously observed defect upon the nuclear depletion of Ysh1 (Baejen et al., 2017; Figure 6B, dark gray). Thus, the latent endonuclease activity of CPF^ΔMpe1^ that we observe *in vitro* is largely insufficient to promote timely RNAPII termination *in vivo*.

The magnitude and apparent length of readthrough transcription varied depending on the local orientation of genes. A termination defect in *KAR2*, for example, produced readthrough transcripts that appeared to invade the downstream codirectional *HCA4* gene and terminated within the next gene, *BET4*, located on the opposite strand (Figure 6A). To investigate whether there was a relationship between the changes in nascent RNA signal and transcription orientation, we calculated the strand-specific log_2_-fold change in the nascent RNA signal for every gene and compared it with the change in RNA signal on the corresponding position in the opposite strand. We also performed a similar analysis using a sliding 1-kb window across the genome. In both cases, the changes in RNA synthesis upon Mpe1 depletion are anticorrelated between strands (Figures 6C and S6F). Thus, readthrough transcription negatively impacts ongoing transcription on the opposite strand, consistent with transcription interference (Shearwin et al., 2005).

To specifically analyze transcription interference, we carried out a *χ*^2^ test of independence between the impact of Mpe1 depletion on nascent RNA and gene organization. This analysis revealed a positive association between increased nascent RNA levels and codirectionally oriented pairs of genes (Figure S6G). This is likely due to readthrough defects increasing the nascent RNA signal of the downstream gene in a codirectional pair (i.e., *HCA4*; Figure 6A). The decrease in nascent RNA was instead associated with genes that share both a convergent and divergent partner. Thus, convergently oriented pairs of genes have an antagonistic impact on each other upon Mpe1 depletion.

To analyze the readthrough transcription variation across the genome, we performed k-means clustering of the strand-specific nascent RNA signal at the 3′ end of convergent gene pairs (Achar et al., 2020). Transcription readthrough occurs beyond the poly(A) site in all the clusters on both strands (minus and plus) in the Mpe1-depleted cells (Figure 6D). The level of readthrough is proportional to the signal preceding the poly(A) site, indicating that the role of Mpe1 is independent of the baseline level of nascent RNA synthesis and is thus globally required for transcription termination.

By comparing the log_2_-fold change between the convergent gene pairs within each cluster, we found that genes with lower baseline transcription levels were more likely to show a decrease in nascent RNA than their counterpart (Figure 6E; plus strand in cluster 1 and minus strand in cluster 3). By contrast, there is an equal impact when baseline RNA synthesis is comparable between both genes in a convergent pair (cluster 2). Given that the level of readthrough is correlated with the baseline level of RNA synthesis, transcription interference and RNAPII collision events are likely skewed against the genes with lower baseline transcription levels.

To specifically evaluate the role of the Mpe1 PSR *in vivo*, we complemented the Mpe1-mAID strain with WT Mpe1, Mpe1^P215G^, or an empty plasmid control. WT Mpe1 rescued the growth defect of the Mpe1 degron strain, but the cells expressing Mpe1^P215G^ had severely restricted growth (Figure S7A). RT-qPCR revealed transcription readthrough after Mpe1 depletion in the empty plasmid control but not when WT Mpe1 was expressed (Figure S7B). Transcription readthrough was also evident in the cells expressing Mpe1^P215G^, but the defect was not as severe as that in the empty plasmid control. Thus, the PSR specifically contributes to Mpe1’s role in transcription termination.

Overall, our genomic analyses reveal that Mpe1 plays a global and essential role in the timely activation of CPF cleavage activity, in transcription termination, and in preventing transcription interference of neighboring genes.

## Discussion

The 3′ end processing machinery couples the recognition of conserved sequence elements in the nascent pre-mRNA with cleavage and polyadenylation. Here, we reveal that Mpe1 plays an important role in efficient cleavage, polyadenylation, and transcription termination. Specifically, we show that (1) Mpe1 contacts RNA and Pfs2 within the polymerase module. The residues that interact with RNA and Pfs2 are also required for endonuclease activation and regulated polyadenylation. (2) Cft2 antagonizes the docking of Mpe1 onto polymerase module. Mpe1 and Cft2 both provide a direct link between the nuclease and polymerase modules. (3) An Mpe1-Ysh1 interaction stably tethers Mpe1 on CPF in the absence of RNA and is essential for Ysh1 activation. (4) Mpe1 is essential for timely transcription termination across the genome.

### PAS recognition by Yth1 is sensed by Mpe1

In the Mpe1-bound structure, the A_2_ base of the PAS is contacted directly by the conserved P215 in Mpe1 through a CH-π interaction. A CH-π interaction is a relatively weak hydrogen bond between a partially charged proton and the delocalized electron π system of an aromatic group (Chakrabarti and Samanta, 1995; Nishio et al., 2014). In principle, CH-π interactions can involve protons from almost any amino acid. Thus, the conservation of a proline at this position may indicate that it is playing a dual role: first, it acts as a general sensor of RNA binding by Yth1 through a CH-π interaction and second, it enforces the correct fold of the Mpe1 PSR due to its sterically restricted side chain. The P215-RNA contact appears to stabilize the PSR of Mpe1 on the polymerase module and is likely involved in “sensing” when CPF is bound to RNA.

### Efficient CPF activation is essential for safeguarding the transcriptome

Our transcriptomic studies show that Mpe1 is globally required for the efficient activation of cleavage activity and, consequently, timely transcription termination. CPF activity may be particularly important in preventing head-to-head RNAPII collisions between convergent genes in yeast (Hobson et al., 2012). Moreover, because intergenic regions in the yeast genome are relatively short compared with mammalian genomes (Chen et al., 2012; Xu et al., 2009), CPF must be activated as soon as the PAS is recognized. In human cells, closely arranged convergent genes show transcription interference upon the depletion of the CPSF endonuclease CPSF73 (Eaton et al., 2020), and the mutations in the β-globin 3′ UTR that cause β-thalassemia lead to termination defects and transcription interference on the downstream gene (Proudfoot, 1986; Whitelaw and Proudfoot, 1986). Thus, in addition to 3′ end processing, CPF/CPSF universally safeguards the transcriptome from unwanted transcription interference.

### RBBP6 may also activate cleavage in human CPSF

The human homolog of Mpe1, RBBP6, is known to play a role in 3′ end processing and has a similar domain organization to Mpe1 (UBL, zinc knuckle, and RING finger) with a metazoan-specific C-terminal extension containing p53 and retinoblastoma-binding sites (Di Giammartino et al., 2014; Pugh et al., 2006). There is a high degree of conservation between yeast Mpe1 and human RBBP6 in the PSR loop that contacts RNA, and recent data show that RBBP6 likely plays a similar role in humans (Boreikaite et al., 2022; Schmidt et al., 2021). In the human mPSF, however, CPSF30 occupies the hydrophobic pocket on WDR33 where the RBBP6 helix would bind (Zhang et al., 2020). It, therefore, seems likely that RBBP6 senses RNA binding in the same way as Mpe1, but RBBP6 interaction with WDR33 may differ.

### Model for CPF activation

**Figure 7:**
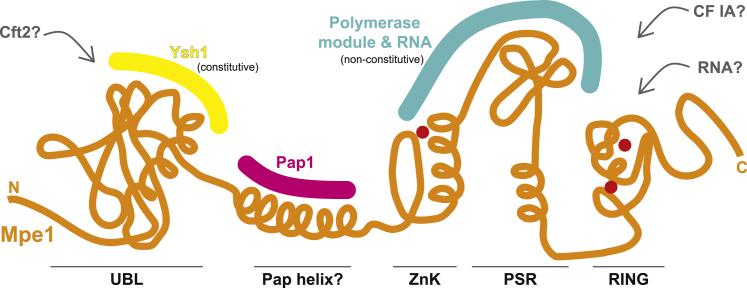
Mpe1 plays a central role in cleavage, polyadenylation, and transcription termination A schematic diagram of Mpe1 is shown (orange) with interactions depicted above. See also Figure S7

Based on these studies, we propose that Mpe1 plays a central role in cleavage, polyadenylation, and transcription termination. Interestingly, all domains of Mpe1 (UBL, zinc knuckle, PSR, and RING) are important for its function (this work; Lee and Moore, 2014; Vo et al., 2001; Figure 7). Prior to RNA binding, Mpe1 may be flexibly tethered to CPF through its interaction with Ysh1, with Cft2 preventing it from docking onto the polymerase module. In this configuration, the endonuclease is likely inactive and too far from the RNA-binding site (Hill et al., 2019; Zhang et al., 2020). This prevents precocious activation of cleavage.

When Yth1 recognizes a newly transcribed PAS in the nascent transcript, the Mpe1 PSR may sense the bound RNA and dock onto the polymerase module. It is possible that a rearrangement of Mpe1 licenses the assembly of CPF, CF IA, CF IB, and RNA into a configuration that is activated for cleavage, for example, by repositioning and remodeling Ysh1. Other CPF subunits may stabilize the simultaneous binding of Cft2 and the Mpe1 PSR to the polymerase module.

Previous studies suggested that Mpe1 and the polymerase module directly interact with CF IA (Casañal et al., 2017; Lee and Moore, 2014; Vo et al., 2001), which is required for activation of cleavage (Hill et al., 2019). It is possible that the docking of Mpe1 onto the polymerase module forms a new interaction surface for CF IA. Interestingly, RBBP6 also interacts with CstF64, the human homolog of the CF IA subunit, Rna15 (Di Giammartino et al., 2014).

Mpe1 also regulates polyadenylation, so it is possible that PSR binding to the polymerase module stabilizes a postcleavage complex. Recent work using structure prediction methods identified a putative interaction between Mpe1 and Pap1 (Humphreys et al., 2021; Figures S7C–S7E), where a semi-conserved helix in Mpe1 is predicted to bind within a groove in Pap1. This could play a role in positioning and regulating Pap1 activity. We speculate that the remodeling of CPF upon RNA recognition could also activate Glc7 in the phosphatase module to promote transcription termination (Schreieck et al., 2014).

In summary, several factors ensure the fidelity of 3′ cleavage and polyadenylation. Conformational transitions may control mRNA 3′ end processing, prevent inappropriate activation of cleavage, and control the length of the poly(A) tail. We show here that Mpe1 may sense when CPF recognizes a PAS sequence. Together, these data suggest that, similar to splicing, recognition and enzymatic processing of an RNA substrate governs CPF remodeling from an inactive to an active state to control mRNA processing.

### Limitations of the study

Our cryo-EM studies were performed with the polymerase module only—other subunits of CPF, CF IA, and CF IB were not present. Thus, we cannot deduce the potential roles of other CPF subunits and/or CF IA and CF IB in the function of Mpe1. It is possible that other proteins also interact with the PSR to regulate cleavage and polyadenylation. In addition, we used a truncated RNA that ends at the cleavage site. The RNA downstream of the cleavage site may also play an important role in activating cleavage. We are, therefore, unable to differentiate whether our structure represents a precleavage or postcleavage complex or whether it contains common features of both.

The only residue of Mpe1 that contacts RNA directly in our structure is a proline. Because prolines are unique in generating a kink in the backbone, mutational studies cannot differentiate between a structural role (the loss of the kink) and a functional role (CH-π interaction with RNA). Nevertheless, the W257A/Y260A mutation in the PSR that disrupts the binding to Pfs2 has the same functional consequences as the P215G mutation, even though they act through different mechanisms.

## STAR★Methods

### Key resources table

**Table:** 

REAGENT or RESOURCE	SOURCE	IDENTIFIER
**Antibodies**		

anti-mAID	MBL International	Cat# M214-3, RRID:AB_2890014
anti-GAPDH-HRP	Thermo Fisher Scientific	Cat# MA5-15738-HRP, RRID:AB_2537659

**Bacterial and virus strains**		

*E. coli DH10* EMBacY	Geneva Biotech	N/A
E. coli TOP10	Thermo Fisher	Cat# C404010

**Chemicals, peptides, and recombinant proteins**		

Auxin (3-Indoleacetic acid)	Sigma	Cat# I3750-100G-A
4-thiouracil	Sigma	Cat# 440736–1G
5-FOA	Zymo Research	Cat# F9001-5
G418	Sigma	Cat# A1720-5G
BioLock	IBA-Lifesciences	Cat# 2-0205-050
Strep-Tactin resin	IBA-Lifesciences	Cat# 2-1201-025
Desthiobiotin	IBA-Lifesciences	Cat# 2-1000-005
Sulfo-SDA (sulfosuccinimidyl 4,4′-azipentanoate)	Thermo Fisher	Cat# 26173
Instant Blue	Abcam	Cat# 119211
Protease inhibitor tablets	Roche	Cat# 11836153001
TRI reagent	Thermo Fisher	Cat# AM9738
DnaseI (Rnase free)	New England Biolabs	Cat# M0303S
EZ-Link HPDP Biotin	Thermo Fisher	Cat# A35390
Dynabeads MyOne Streptavidin C1	Thermo Fisher	Cat# 65001
FuGENE HD	Promega	Cat# E2311
UltraPure Salmon sperm DNA solution	Invitrogen	Cat# 15632011
LDS Sample Buffer	Pierce	Cat# 84788
ECL Western Blotting Reagents	Cytiva	Cat# RPN2106
GlycoBlue Coprecipitant	Thermo Fisher	Cat# AM9515
Phenol:Chloroform:Iso-amyl alcohol (125:24:1)	Sigma	Cat# P1944-100ML
Recombinant protein: *S. cerevisiae* polymerase module	(Casañal et al., 2017)	N/A
Recombinant protein: *S. cerevisiae* polymerase module-Mpe1-SII	This study	N/A
Recombinant protein: *S. cerevisiae* polymerase module-Mpe1^P215G^-SII	This study	N/A
Recombinant protein: *S. cerevisiae* polymerase module-Mpe1^W257A/Y260A^-SII	This study	N/A
Recombinant protein: *S. cerevisiae* polymerase module-Mpe1-pc*CYC1*	This study	N/A
Recombinant protein: *S. cerevisiae* polymerase module-Mpe1-Cft2(S)-pc*CYC1*	This study	N/A
Recombinant protein: *S. cerevisiae* polymerase module-Mpe1-yPIM-pc*CYC1*	This study	N/A
Recombinant protein: *S. cerevisiae* CPF	(Kumar et al., 2021), This study	N/A
Recombinant protein: *S. cerevisiae* nuclease-phosphatase modules (nuc-phos)	(Kumar et al., 2021)	N/A
Recombinant protein: *S. cerevisiae* Ysh1-Cft2-phosphatase module	This study	N/A
Recombinant protein: *S. cerevisiae* CPF^Δ^^M^^pe1^	This study	N/A
Recombinant protein: *S. cerevisiae* CPF^P215G^	This study	N/A
Recombinant protein: *S. cerevisiae* CPF^W257A/Y260A^	This study	N/A
Recombinant protein: *S. cerevisiae* CF IA	(Kumar et al., 2021)	N/A
Recombinant protein: *S. cerevisiae* CF IB	(Hill et al., 2019)	N/A
yPIM (peptide sequence): ASKHKMFPFNPAKIKKDDYGTVVDFTMFLPDDS	This study (GenScript)	N/A

**Critical commercial assays**		

NEBNext Ultra II Directional RNA Library Prep Kit for Illumina	New England Biolabs	Cat# E7760S
NEBNext rRNA depletion kit	New England Biolabs	Cat# E6310S
Power SYBR Green PCR	Thermo Fisher	Cat# 4367659
Phusion high-fidelity DNA Polymerase	New England Biolabs	Cat# M0530S
HiScribe T7 Quick High Yield RNA Synthesis kit	New England Biolabs	Cat# E2050S
Monarch RNA Cleanup kit	New England Biolabs	Cat# T2030S
RNA 6000 Nano Kit	Agilent	Cat# 5067-1511
SuperScriptIII Reverse Transcriptase	Invitrogen	Cat# 18080-093

**Deposited data**		

RNA-seq of total and nascent RNA	This study	ArrayExpress: E-MTAB-10820
RNA-seq after nuclear depletion of Ysh1	(Baejen et al., 2017)	GEO: GSE79222
Cross-linking mass spectrometry data	This study	ProteomeXchange: PXD027482
Original images, chromatograms and qPCR data	This study	Mendeley Data: https://dx.doi.org/10.17632
Polymerase module-Mpe1-RNA (EM map)	This study	EMDB: EMD-14710
Polymerase module-Cft2(S) (EM map)	This study	EMDB: EMD-14711
Polymerase module-Mpe1-yPIM-RNA (EM map)	This study	EMDB: EMD-14712
Polymerase module-Mpe1-RNA (model)	This study	PDB: 7ZGP
Polymerase module-Cft2(S) (model)	This study	PDB: 7ZGQ
Polymerase module-Mpe1-yPIM-RNA (model)	This study	PDB: 7ZGR
Polymerase module	(Casañal et al., 2017)	EMDB: 3908
Polymerase module	(Casañal et al., 2017)	PDB: 6eoj
mPSF-PIM	(Zhang et al., 2020)	PDB: 6urg
Pap1-Fip	(Meinke et al., 2008)	PDB: 3c66

**Experimental models: Cell lines**		

*Sf9*	Oxford Expression Technologies Ltd.	Cat# 600100-Sf9 cells

**Experimental models: Organisms/strains**		

*S. cerevisiae*: MATa ade2-1 his3-11,15 trp1-1 leu2-3,112 can1-100 ura3-1∷ADH1-OsTIR1(pMK200, URA3)	(Nishimura and Kanemaki, 2014)	YMK728 (S2-31)
*S. cerevisiae*: YMK728 Mpe1-3miniAID-3FLAG	This study	JRY101 (S2-37)
*S. cerevisiae*: JRY101 pRS314	This study	JRY200 (S3-64)
*S. cerevisiae*: JRY101 pRS314-MPE1	This study	JRY208 (S4-24)
*S. cerevisiae*: JRY101 pRS314-mpe1(P215G)	This study	JRY210 (S4-26)
*S. cerevisiae*: YMK728 Mpe1-3mAID-3FLAG (OsTIR1-, URA3-)	This study	JRY114 (S2-52)
*S. pombe*: h+	Juan Mata	JU60 (S3-30)

**Oligonucleotides**		

Complete list of DNA oligonucleotide sequences	This study	Table S1
precleaved *CYC1* (pc*CYC1*): 5ʹ 6-FAM-UUUAUAGUUAUGUUAGUAUUAAGAA CGUUAUUUAUAUUUCAA 3′	(Casañal et al., 2017)	N/A
*CYC1*: 5ʹ 6-FAM-UUUAUAGU UAUGUUAGUAUUAAGAACGUUAUUUAU AUUUCAAAUUUUUCUUUUUUU-A647 3′	(Hill et al., 2019)	*CYC1a*

**Recombinant DNA**		

pRS314	(Sikorski and Hieter, 1989)	P19-17
pRS314-Mpe1	This study	P34-48
pRS314-Mpe1(P215G)	This study	P34-49
pACEBac1-Mpe1(FDRP)-TEV-SII	This study	P24-58
(modified) pBig1A	(Hill et al., 2019; Weissmann et al., 2016)	P24-63
(modified) pBig1B	(Hill et al., 2019; Weissmann et al., 2016)	P24-64
(modified) pBig2AB	(Hill et al., 2019; Weissmann et al., 2016)	P25-3
pACEBac1-Cft1	(Casañal et al., 2017)	P14-39
pACEBac1-Pfs2-SII	(Casañal et al., 2017)	P14-40
pACEBac1-Yth1	(Casañal et al., 2017)	P14-42
pACEBac1-Mpe1(P215G)-TEV-SII	This study	P31-24
pACEBac1-Mpe1(W257A, Y260A)-TEV-SII	This study	P31-25
pACEBac1-Cft2-SII	(Hill et al., 2019)	P25-7
pACEBac1-Cft2(F537A, Y549A, F558A)-TEV-SII	This study	P34-45
pACEBac1-Mpe1 (ZnK-PSR)-TEV-SII	This study	P27-60
pACEBac1-Mpe1^ΔPSR^-TEV-SII	This study	P34-47
pACEBac1-Mpe1^ΔZnK^-TEV-SII	This study	P34-46
pIDC-Fip1	(Casañal et al., 2017)	P14-44
pIDC-Pap1	(Casañal et al., 2017)	P14-45
pIDS-Mpe1-SII	(Hill et al., 2019)	P14-56
pIDS-Ysh1	(Hill et al., 2019)	P14-59
pIDS-Cft2	(Hill et al., 2019)	P14-57
pBig1A-Cft1-Mpe1-SII	This study	P25-38
pBig1A-Pap1-Mpe1-SII	This study	P25-39
pBig1A-Pfs2-Mpe1-SII	This study	P25-40
pBig1A-Fip1-Mpe1-SII	This study	P25-41
pBig1A-Yth1-Mpe1-SII	This study	P25-42
pBig1A-Construct A (Cft1-Pap1-Pfs2-Fip1-Yth1)	(Hill et al., 2019)	P20-1
pBig1B-Mpe1-SII	This study	P25-41
pBig1A-Construct B (Cft1-Pap1-Pfs2-SII-Fip1-Yth1)	(Hill et al., 2019)	P20-3
pBig2AB-Construct A + Mpe1-SII	This study	P26-20
pBig1B-Construct AX (Ysh1-Cft2)	This study	P20-54
pBig2AB-Ssu72-Pti1-Glc7-Ref2-SII-Swd2	(Kumar et al., 2021)	P27-37
pET28a +(modified) 6H-3C-Cft2(short)	Chris Hill	P19-8

**Software and algorithms**		

Integrated Genome Viewer (v. 2.4.11)	(Robinson et al., 2011)	https://software.broadinstitute.org/software/igv/
RUV-seq (v. 1.20.0)	(Risso et al., 2014)	https://bioconductor.org/packages/release/bioc/html/RUVSeq.html
Rsubread (v. 2.0.1)	(Liao et al., 2014)	https://bioconductor.org/packages/release/bioc/html/Rsubread.html
STAR (v. 2.6.0a)	(Dobin et al., 2013)	https://github.com/alexdobin/STAR
TrimGalore (v. 0.4.5)	https://www.bioinformatics.babraham.ac.uk/projects/trim_galore/	https://github.com/FelixKrueger/TrimGalore
SAMtools	(Li et al., 2009)	http://samtools.sourceforge.net/
Deeptools (v. 3.1.3)	(Ramirez et al., 2016)	https://deeptools.readthedocs.io/en/develop/
R (v. 3.6.0)	(R Core Team, 2019)	https://www.r-project.org
DESeq2 (v. 1.26.0)	(Love et al., 2014)	https://bioconductor.org/packages/release/bioc/html/DESeq2.html
SeqPlots	(Stempor and Ahringer, 2016)	https://github.com/Przemol/seqplots
Prism 8 (v. 8.1.2)	N/A	https://www.graphpad.com
cryoEF	(Naydenova and Russo, 2017)	https://www.mrc-lmb.cam.ac.uk/crusso/cryoEF/
ProtParam	(Gasteiger, 2005)	https://web.expasy.org/protparam/
Relion 3.1	(Zivanov et al., 2018)	https://github.com/3dem/relion
DynamX	Waters	N/A
Coot (v. 0.9.5.1-pre)	(Emsley and Cowtan, 2004; Emsley et al., 2010)	https://www2.mrc-lmb.cam.ac.uk/personal/pemsley/coot
ChimeraX (v. 1.2)	(Goddard et al., 2018)	https://www.cgl.ucsf.edu/chimerax/
PDBePISA	(Krissinel and Henrick, 2007)	https://www.ebi.ac.uk/pdbe/pisa/
ClustalW	(Goujon et al., 2010; Sievers et al., 2011)	https://www.ebi.ac.uk/Tools/msa/clustalo/
ImageJ (v. 1.52a)	(Schneider et al., 2012)	https://imagej.net/software/imagej/
Jalview (v 1.0)	(Clamp et al., 2004)	http://www.compbio.dundee.ac.uk/ftp/embnet.news/vol5_4/embnet/body_jalview.html

**Other**		

Insect-XPRESS™ Protein-free Insect cell medium	Lonza	Cat# BELN12-730Q
MonoQ 5/50 GL	Cytiva	Cat# 17516601
HiTrap Heparin 1ml	Cytiva	Cat# 17040601
HiLoad 16/600 Superdex 200 pg	Cytiva	Cat# 28989335
UltrAuFoil R 1.2/1.3 on Au 300 mesh grids	Quantifoil	Cat# N1-A14nAu30-50
Superose 6 Increase 3.2/300	Cytiva	Cat# 29091598
Superdex 30 Increase 3.2/300	Cytiva	Cat# 29219758

### Resource availability

#### Lead contact

Further information and requests for resources and reagents should be directed to and will be fulfilled by the lead contact, Lori Passmore (passmore@mrc-lmb.cam.ac.uk).

#### Materials availability

All unique/stable reagents generated in this study are available from the lead contact with a completed Materials Transfer Agreement.

### Experimental model and subject details

All gene cloning, manipulation and plasmid propagation steps involving pACEBac1, pBIG1 or pBIG2 series vectors were carried out in *Escherichia coli* DH5a or TOP10 cells grown in 2 X TY or LB media supplemented with appropriate selection antibiotics. *E.coli* DH10 EMBacY cells were used for bacmid isolation.

Recombinant Cft2(S) was expressed in *E. coli* BL21 Star (DE3) cells or BL21 Star (DE3) pLysS cells grown in 2 X TY media until an OD_600nm_ of 0.6 – 1.0 was reached. Expression was induced with 0.5 mM IPTG for an appropriate time and temperature as described. For all other recombinant proteins and complexes, the *Spodoptera frugiperda* Sf9 cell line was used for baculovirus-driven overexpression. Suspension cultures were grown at 27°C, 140 rpm in Insect-XPRESS protein-free insect cell medium with L-glutamine (Lonza).

Functional studies were performed in *Saccharomyces cerevisiae* strains listed in the key resources table. Yeast strains were grown at 30°C with shaking at 180 rpm in YPD media (YPD media per L: 20 g peptone, 20 g D-glucose, 10 g yeast extract). Synthetic complete or drop out media was used as indicated. Media was supplemented with appropriate selection antibiotics.

### Method details

#### Cloning

Oligonucleotides and plasmids used in this study are listed in Table S1 and the key resources table, respectively.

##### Mpe1 with polymerase module subunits

Twin strep (SII)-tagged Mpe1 was amplified from pIDS-Mpe1 (Hill et al., 2019) using primers pIDS_CasII_F and pIDS_Casω-R. *E. coli* codon-optimized genes encoding Cft1, Pap1, Pfs2, Fip1 and Yth1 (GeneArt) were amplified using primers pB/pIDC_CasI_F and pB/pIDC_CasI_R. Mpe1 and each of the polymerase module genes were cloned into a SwaI (NEB) digested pBig1a vector of a modified biGBac system (Hill et al., 2019; Weissmann et al., 2016) via Gibson assembly. Colonies were screened for correct constructs by restriction digest using BamHI and XbaI (NEB).

##### Polymerase module-Mpe1

pBig1a carrying genes encoding subunits of the polymerase module (Cft1, Pap1, Pfs2, Fip1 and Yth1) (Kumar et al., 2021) and pBig1b carrying Mpe1-3C-SII were digested with PmeI (NEB) to release the gene cassettes. Cassettes were cloned into PmeI-digested pBig2ab via Gibson assembly and transformed into chemically competent Top10 *E. coli*. Constructs were screened via SwaI (NEB) restriction digestion for insertion of all genes.

##### Mpe1 variants

The Mpe1^FDRP^ variant (F9A, D45K, R76E, P78G) was generated by Gibson assembly of PCR products using the corresponding primers listed in Table S1. PCR products have overlapping sequences to allow for Gibson assembly into the pMA vector. Mpe1^FDRP^ was subcloned via PCR and Gibson assembly into a modified pACEBac1 vector that introduces an in-frame TEV cleavage site followed by an SII tag at the 3ʹ-end of the insert (pACEBAC_TEV_SII). Mpe1^P215G^, Mpe1^W257A/Y260A^, Mpe1^ΔZnK^ and Mpe1^ΔPSR^ variants were similarly generated with PCR products carrying the respective mutations and cloned by Gibson assembly into a BamHI/XhoI (NEB) digested pACEBAC_TEV_SII vector. Constructs were screened and mutations confirmed by Sanger sequencing (Source Biosciences) using the pACE_Mpe1_F primer.

##### Cft2 yPIM variant

The Cft2^mut1^ construct (F537A, Y549A, F558A) was generated by amplifying two Cft2 fragments with overlapping overhangs from pIDS-Cft2 (Hill et al., 2019) using the corresponding primers listed in Table S1. Fragments were assembled into pACEBAC_TEV_SII by Gibson assembly.

##### Ysh1-Cft2 construct

Genes encoding Ysh1 and Cft2 were amplified from pIDS-Ysh1 and pIDS-Cft2 (Hill et al., 2019) using pIDS_CasI_F and pIDS_CasI_R (for Cft2), and pIDS_CasII_F and pIDS_Casω_R (for Ysh1). PCR products were cloned into SwaI-digested pBig1B by Gibson assembly. This construct was used for co-infections with phosphatase module or polymerase module (see below).

#### Baculovirus-mediated protein overexpression

Bacmids were constructed by transforming pACEBac1 or pBig constructs into *E. coli* EMBacY cells. Correct integration into the baculovirus genome was screened with blue/white selection using X-gal. Bacmids were prepared from 5 ml overnight cultures of selected white colonies using the QIAprep miniprep kit protocol (Qiagen). Isopropanol precipitation of the bacmid DNA was performed instead of on-column purification. *Sf9* cells (3-6 wells with 2 ml of *Sf9* cells at 0.5 x10^6^ cells/ml) were transfected with 10 μg/well of purified bacmid DNA and the FuGENE HD transfection reagent. The P1 virus was prepared from the supernatant media of transfected cells (after 3-4 days and visualization of YFP-expressing cells) by filtering the supernatant and adding 1 volume of fetal bovine serum. P2 virus was prepared by infecting 25 ml of *Sf9* cells (2x10^6^ cells/ml) with a final 1:50 dilution of the P1 virus. Cells were diluted with fresh media until doubling arrested (2-3 days). Cells were harvested and the supernatant filtered when cells were YFP positive and viability was within 80%-90%. For large-scale expression, a final 1:100 dilution of the P2 virus was used to infect 3-6 L of *Sf9* cells (2x10^6^ cells/ml) for 2-3 days (Hill et al., 2019; Kumar et al., 2021).

##### Co-infections

For the complex of the combined Ysh1-Cft2-phosphatase module, 3 L of *Sf9* cells at 2x10^6^ cells/ml were co-infected (1:1 volume ratio) with two viruses; one carrying genes encoding Ysh1 and Cft2 and another carrying genes encoding subunits of the phosphatase module with an SII tag on the Ref2 subunit (Kumar et al., 2021).

For the polymerase module-Cft2 complex, *Sf9* cells were co-infected with two viruses (in a 1:1 ratio); one carrying genes encoding the polymerase module (with an SII tag on Pfs2) and another carrying untagged Cft2 and Ysh1.

#### Pulldowns

25 ml of *Sf9* at 2x10^6^ cells/ml cells were infected with P2 viruses carrying Mpe1-SII paired with Cft1, Pap1, Pfs2, Fip1 or Yth1 for 48 hours. Cell pellets were lysed in Buffer A (50 mM HEPES pH 8, 75 mM NaCl, 1 mM TCEP), and cleared lysate mixed with 40 μl of Strep-Tactin slurry (IBA, cat. No. 2-1201-025) equilibrated in Buffer A. Protein-bound resin was washed with Buffer A and eluted with 40 μl Buffer A containing 1.2 mg/ml desthiobiotin (IBA, cat. No. 2-1000-005). Eluates were analyzed on SDS-PAGE stained with Instant Blue (Abcam, cat. no. 119211).

#### Protein purification

For protein purification from *Sf9* cells the indicated lysis buffer (see below) was supplemented with 1 ml of BioLock (IBA, cat. No. 2-0205-050) and 3x protease inhibitor tablets (Roche, cat. No. 11836153001). 200 ml lysis buffer was used to resuspended frozen cell pellets. Cells were lysed by sonication at 4°C using the VC 750 ultrasonic processor (Sonics) with a 10 mm tip (5 seconds on, 10 seconds off at 50% amplitude for 3 minutes). Lysate was cleared at 18,000 rpm for 20 min at 4°C using a JA 25.50 rotor. The cleared supernatant was incubated for 1-2 hours with 2-5 ml of StrepTactin slurry equilibrated in the respective lysis buffer. RNase and DNase were omitted from the lysis buffer (Casañal et al., 2017; Hill et al., 2019; Kumar et al., 2021).

For polymerase module-Mpe1, a frozen pellet of insect cells (3-6 L at 2x10^6^ cells/ml) expressing polymerase module and SII-tagged Mpe1 were lysed in Buffer A supplemented with 3x protease inhibitor tablets (Roche, cat. No. 11836153001) and 1 ml BioLock. Cleared lysate was applied to StrepTactin resin and washed with Buffer A. Protein was eluted with 20 ml of Buffer A supplemented with 1.2 mg/ml desthiobiotin. Eluate was filtered through a 0.45 μm filter and applied to a 1 ml MonoQ 5/50 GL column equilibrated in Buffer A. The complex was eluted off the column using a linear gradient up to 50% Buffer B (50 mM HEPES pH 8, 1 M NaCl, 1 mM TCEP) over 40 column volumes. During elution, Mpe1 and polymerase module dissociate, elute as distinct peaks, and were kept separate following the purification. Individual fractions containing Mpe1 or polymerase module were pooled and concentrated using a 30 kDa or 100 kDa cut-off concentrator (Amicon), respectively. Concentration was determined using a nanodrop (ThermoFisher) and the theoretical extinction coefficient for Mpe1 (32,430 M^-1^ cm^-1^) or polymerase module (298,530 M^-1^ cm^-1^) calculated in ProtParam (Gasteiger, 2005). The polymerase module from this purification is untagged. Purified protein was flash frozen in liquid N_2_ and kept at -80°C.

The polymerase module-Cft2 complex was purified as described for the polymerase module-Mpe1 complex from insect cells co-infected with viruses carrying the polymerase module and Ysh1-Cft2. Ysh1 did not stably co-purify with this complex. Concentration was determined using the theoretical extinction coefficient for the polymerase module-Cft2 complex (383,790 M^-1^ cm^-1^) calculated in ProtParam.

SII-tagged Mpe1 variants were purified following the same procedure described for polymerase module-Mpe1 complex with some modifications. Mpe1 was purified from 1-3 L at 2x10^6^ cells/ml of pelleted and frozen insect cells. The filtered eluate from the Strep-Tactin affinity purification was applied to a HiTrap Heparin 1 ml column (Cytiva, cat. No. 17040601).

##### Cft2(*S*)

9 L of BL21 star cells carrying the pET28a +(modified) 6H-3C-Cft2(S) vector were induced with 0.5 mM IPTG overnight at 18°C. Cells were lysed in Buffer C (50 mM HEPES pH 7.9, 30 mM imidazole, 250 mM NaCl, 1 mM TCEP) supplemented with protease inhibitor tablets, DNaseI and RNaseA. Cleared lysate was incubated with Ni-NTA (Qiagen) resin, washed with Buffer C and eluted with Buffer D (50 mM pH 7.9, 500 mM imidazole, 250 mM NaCl). Pooled fractions were cleaved with 3C protease (140 μg/ml) at 4°C. Cleaved Cft2(S) was further purified by anion exchange chromatography using a 1 ml MonoQ 5/50 GL column equilibrated in Buffer E (50 mM HEPES pH 7.9, 150 mM NaCl, 1 mM TCEP) over a gradient up to 100% over 100 column volumes using Buffer F (50 mM HEPES pH 7.9, 1 M NaCl, 1 mM TCEP). Pooled fractions containing Cft2(S) were concentrated using a VivaSpin concentrator (30 kDa cutoff) and further purified by size exclusion chromatography using a HiLoad 16/600 Superdex 200 pg column (Cytiva, 28989335) equilibrated in Buffer E. Fractions containing Cft2(S) were pooled and concentrated as before and flash frozen in liquid N_2_ and stored at -80°C.

##### Ysh1-Cft1-phosphatase

For purification of combined Ysh1-Cft2-phosphatase module complex, cells were lysed in Buffer G (50 mM HEPES pH 8, 150 mM KCl, 0.5 mM Mg(OAc)_2_, 0.5 mM TCEP) supplemented with 3x protease inhibitor tablets and 1 ml BioLock. Protein-bound resin was washed with Buffer G and eluted from the StrepTactin resin with Buffer G containing 1.2 mg/ml desthiobiotin. Eluate was filtered through a 0.45 μm filter and applied to a 1 ml MonoQ 5/50 GL column equilibrated in Buffer G. The complex was eluted off the column using a linear gradient up to 50% Buffer H (50 mM HEPES pH 8, 1 M KCl, 0.5 mM Mg(OAc)_2_, 0.5 mM TCEP) over 40 column volumes. Fractions with eluted protein showing correct stoichiometry were pooled, concentrated in an Amicon concentrator (100 kDa cutoff) and flash frozen. Concentration was determined using the theoretical extinction coefficient for Ysh1-Cft2-phosphatase module (334,400 M^-1^ cm^-1^) calculated in ProtParam.

##### Cft2 and Cft2^mut1^

3 L of *Sf9* cells expressing either Cft2-SII or Cft2^mut1^-SII were lysed using Buffer I (50 mM HEPES pH 8, 150 mM NaCl, 0.5 mM MgCl_2_, 1 mM TCEP) supplemented with 4x protease inhibitor tablets and 1 ml BioLock. Proteins were affinity purified with Strep-Tactin resin and eluted with desthiobiotin as described above. Eluate was purified using a HiTrap Heparin 1 ml column with a linear 0-100% gradient of Buffer F. Protein was concentrated with using an Amicon concentrator (30 kDa cutoff) and further purified on a Hi-Load 16/600 Superdex 200pg using SEC 300 (50 mM HEPES pH 8, 300 mM NaCl, 0.5 mM TCEP). Protein concentration was determined using the theoretical extinction coefficient of Cft2 (85,260 M^-1^ cm^-1^) calculated using ProtParam.

##### *In vitro* reconstitutions

Purified CPF complexes were made by mixing 5 μM polymerase module, 5 μM Ysh1-Cft2-phosphatase module and 15 μM Mpe1 or any variants thereof. The final volume was brought to 50 μl with SEC 150 KCl buffer (20 mM HEPES pH 8, 150 mM KCl, 0.5 mM Mg(OAc)_2_, 0.5 mM TCEP). Sample was kept on ice before being loaded on a Superose 6 Increase 3.2/300 (Cytiva, cat. No. 29091598) equilibrated in SEC 150 KCl buffer. For polymerase module complexes to be used in EMSAs or polyadenylation assays, 10 μM of polymerase module was mixed with 30 μM Mpe1 or variants thereof and purified using a Superose 6 Increase 3.2/300 column with SEC 50 buffer (20 mM HEPES pH 8, 50 mM NaCl, 0.5 mM TCEP). Fractions showing all components of the respectively assembled complexes with correct stoichiometry were pooled, concentrated in an Amicon concentrator (100 kDa cutoff) and flash frozen. Concentration was determined using the theoretical extinction coefficient for polymerase module-Mpe1 (330,960 M^-1^ cm^-1^), CPF^ΔMpe1^ (632,930 M^-1^ cm^-1^) or CPF (665,360 M^-1^ cm^-1^) calculated in ProtParam. For cryo-EM sample preparation, 3 μl from the peak fraction were used per grid.

##### Analytical size exclusion chromatography

For binding studies, we used the following: 10 μM Cft2(S) or 720 μM synthetic yPIM peptide (GenScript) with 5 μM of polymerase module and 15 μM Mpe1; 5 μM of the polymerase module-Cft2 complex with 15 μM Mpe1; 10 μM Cft2^mut1^ or 6.5 μM WT Cft2 with 5 μM polymerase module. In the cases where RNA was included, 10 μM of 5ʹ FAM-labeled precleaved *CYC1* substrate (IDT) or an unlabeled *in vitro* transcribed precleaved *CYC1* substrate (key resources table) was added to the complex as indicated. The final volume of the assembly was brought to 50 μl with SEC 50 buffer. In the case of the mutant yPIM binding studies, SEC 300 was used instead. All complexes were analyzed using a Superose 6 Increase 3.2/300 using either SEC 300 (for yPIM mutational studies) or SEC 50 (for all other complexes).

For binding studies of the Mpe1^FDRP^ variant to polymerase module, we used 15 μM of Mpe1^FDRP^ and 5 μM of polymerase module and analyzed the interaction with a Superose 6 Increase 3.2/300 column using SEC 50 buffer. To test incorporation of Mpe1^FDRP^ into CPF we used 15 μM of Mpe1^FDRP^, 5 μM of polymerase module and 5 μM of Ysh1-Cft2-phosphatase module and analyzed the interaction with a Superose 6 Increase 3.2/300 column using SEC 150 KCl buffer.

#### *In vitro* transcription

*In vitro* transcribed precleaved *CYC1* RNA was generated using the HiScribe T7 Quick High Yield RNA Synthesis kit scaled up 3x and following the manufacturer instructions. The template was prepared by mixing 20 μl of 100 μM R00_T7_Fwd and R00_T7_Rev with 10 μl of SEC 300 buffer. The oligos were heated to 95°C for 5 min and slowly cooled to 4°C using a thermocycler (1% ramp). A single RNA product was confirmed by visualizing it on a 20% urea-PAGE gel, and the RNA purified using the Monarch RNA cleanup kit (NEB).

#### Cryo-EM

##### Sample preparation and data collection

1.2/1.3 UltrAuFoil (Quantifoil) grids (Russo and Passmore, 2014) were glow discharged using an Edwards Sputter Coater S150B at setting 8 for 90 sec. Complexes were freshly assembled as described above, and 3 μl from the peak fraction (at 300-500 nM) was used per grid. Grids were frozen in liquid ethane using Vitrobot Mark IV (ThermoFisher) with 5 sec blotting, blotting force -10 at 4°C in 100% humidity. For the polymerase module-Mpe1-RNA complex 11,856 movies were collected on Krios II at eBIC with a K3 detector in counting mode (bin 1), pixel size of 0.83 Å/pixel, total dose of 40 e^-^/Å^2^ with a defocus range of -0.5 μm to -3.1 μm in 0.2 μm steps. For the polymerase module-Mpe1-Cft2(S)-RNA complex 5,118 movies were collected on Krios III at MRC-LMB with a K3 detector in counting mode (bin 1), pixel size of 0.86 Å/pixel, total dose of 36.9 e^-^/Å^2^ with a defocus range of -0.5 μm to -3.1 μm in 0.2 μm steps. For the polymerase module-Mpe1-yPIM-RNA complex 19,524 movies were collected on Krios III at MRC-LMB with a K3 detector in super-resolution counting mode (bin 2), pixel size of 0.86 Å/pixel, total dose of 40 e^-^/Å^2^ with a defocus range of -0.5 μm to -3.1 μm in 0.2 μm steps.

##### Cryo-EM data processing

A general description of cryo-EM data processing is provided below. For complex-specific details regarding the polymerase module-Mpe1-RNA complex, the polymerase module-Mpe1-Cft2(S)-RNA complex or the polymerase module-Mpe1-yPIM-RNA complex please refer to Figures S1H, S3D, or S4B, respectively.

Multi-frame movies from each data collection were processed using Relion 3.1 (Zivanov et al., 2018). Per-micrograph beam-induced motion was estimated and corrected using MotionCor2 using a 5x5 grid (Zheng et al., 2017), and the CTF was estimated using Gctf (Zhang, 2016). Before further processing, the best micrographs were selected, first based on their estimated resolution (at least 5 Å), and then according to their figure of merit (at least 0.05).

We next used the previously-reported polymerase module map (EMDB: EMD-3908) (Casañal et al., 2017), low-pass filtered to 35 Å, for template-based particle picking using Relion 3.1. Particles were first extracted binned ∼5x to the pixel size indicated in the supplementary figure schematics. Depending on the number of particles, they were randomly split into four equally-sized groups and eventually re-grouped. 2D Classification was carried out for each group using the Relion 3.1 implementation, and class averages without clear presence of particles were discarded. 2D classification was repeated as indicated.

Next we used the aforementioned polymerase module map to carry out 3D classification with or without a mask. Classes with isotropic maps and distinct internal features were selected and 3D refined. Refined maps were 3D classified without image alignment and classes selected again based on map isotropy and clear internal features. Particles were then re-extracted to their original pixel size as indicated and 3D refined. CTF refinement and per-particle Bayesian polishing were performed for maps of the polymerase module-Mpe1-RNA and polymerase module-Mpe1-Cft2(S)-RNA using the Relion 3.1 implementation.

##### Model construction, refinement, and analysis

Mpe1 and RNA were manually modelled into their respective densities using Coot [v. 0.9.5.1-pre] (Emsley and Cowtan, 2004; Emsley et al., 2010). The yPIM was modelled using the PIM of CPSF100 (PDB: 6urg) (Zhang et al., 2020) as an initial model and modified in Coot. Models were refined in Phenix Real Space Refine [v. 1.19.2-4158] (Adams et al., 2010) and Coot. Models and maps were further visualized and analyzed in ChimeraX [v. 1.2] (Goddard et al., 2018). Buried surface area was calculated using PDBePISA (Krissinel and Henrick, 2007).

Protein sequences for Cft1, Pfs2, Mpe1 and Yth1 from *Saccharomyces cerevisiae*, *Schizosaccharomyces pombe*, *Danio rerio*, *Homo sapiens*, *Mus musculus*, *Caenorhabditis elegans*, *Drosophila melanogaster*, *Candida albicans* and *Kluyveromyces lactis* were aligned using ClustalW (Goujon et al., 2010; Sievers et al., 2011). The alignment was visualized in Jalview and was used to color the surface of the polymerase module-Mpe1-RNA structure using the entropy-based conservation index of AL2CO (Pei and Grishin, 2001) implemented in ChimeraX. Orientation distribution plots and efficiency of orientation distribution (E_OD_) were calculated using cryoEF (Naydenova and Russo, 2017).

#### Crosslinking mass spectrometry

The crosslinker sulfo-SDA (sulfosuccinimidyl 4,4′-azipentanoate) (Thermo Scientific Pierce) was dissolved in crosslinking buffer (20 mM HEPES, 50 mM NaCl, 0.5 mM TCEP, pH 8.0) to 4 mM before use. For photo-activation, the polymerase module-Cft2(S)-Mpe1-RNA complex (at 0.5 mg/ml in 250 μl) was incubated with sulfo-SDA at a final concentration of 2 mM for 2 h on ice. The sample was irradiated with UV light at 365 nm for 15 min. Crosslinked complex was visualized in Tris-Acetate gels (Thermo, cat. No. EA0375BOX). Gel slices containing the crosslinked complex were cut out with a clean razor blade and dehydrated with LCMS-grade acetonitrile. Samples were subsequently denatured in 8 M urea, 100 mM NH_4_HCO_3_. The proteins were derivatized with iodoacetamide and digested with LysC endoproteinase (Wako) for 4 h at 25 °C. After dilution of the sample to a urea concentration of 1.5 M with 100 mM NH_4_HCO_3_, trypsin (Thermo Scientific Pierce) was added and the samples were digested for 16 h at 25 °C. The resulting tryptic peptides were extracted and desalted using C18 StageTips.

Eluted peptides were fractionated using a Superdex 30 Increase 3.2/300 column (GE Healthcare) at a flow rate of 10 μl/min using 30 % (v/v) acetonitrile and 0.1 % (v/v) trifluoroacetic acid as mobile phase. 50 μl fractions were collected and dried. Five highest molecular weight samples for analysis were resuspended in 0.1 % (v/v) formic acid, 1.6 % (v/v) acetonitrile for LC-MS/MS.

LC-MS/MS analysis was performed on an Orbitrap Fusion Lumos Tribrid mass spectrometer (Thermo Fisher) coupled on-line with an Ultimate 3000 RSLCnano system (Dionex, Thermo Fisher). Samples were separated on a 50-cm EASY-Spray column (Thermo Fisher). Mobile phase A consisted of 0.1 % (v/v) formic acid and mobile phase B of 80 % (v/v) acetonitrile with 0.1 % (v/v) formic acid. Flow rates were 0.3 μl/min using gradients optimized for each chromatographic fraction from offline fractionation, ranging from 2 % to 45 % mobile phase B over 90 min. Mass spec data were acquired in data-dependent mode using the top-speed setting with a three second cycle time. For every cycle, the full scan mass spectrum was recorded using the Orbitrap at a resolution of 120,000 in the range of 400 to 1,600 m/z. Ions with a precursor charge state between 3+ and 7+ were isolated and fragmented. Each precursor was fragmentated by higher-energy Collisional Dissociation (HCD) at 26 %, 28 % and 30 %. The fragmentation spectra were then recorded in the Orbitrap with a resolution of 60,000. Dynamic exclusion was enabled with single repeat count and 60-seconds exclusion duration. A recalibration of the precursor m/z was conducted based on high-confidence (<1 % False Discovery Rate, FDR) linear peptide identifications. The re-calibrated peak lists were searched against the sequences and the reversed sequences (as decoys) of crosslinked peptides using the Xi software suite (v.1.6.745) for identification (Mendes et al., 2019). Final crosslink lists were compiled using the identified candidates filtered to 1 % FDR on link level with xiFDR v.2.1.5.2 (Lenz et al., 2021).

#### Hydrogen-deuterium exchange mass spectrometry

An aliquot of 5 μl of 10 μM polymerase module or polymerase module-Mpe1 complex was incubated with 45 μl of D_2_O buffer at room temperature for 3, 30, 300 and 3000 seconds, and the reaction was quenched and stored at -80 °C. The quenched protein samples were rapidly thawed and subjected to proteolytic cleavage by pepsin followed by reversed phase HPLC separation. Briefly, the protein was passed through an Enzymate BEH immobilized pepsin column, 2.1 x 30 mm, 5 μm (Waters, UK) at 200 μl/min for 2 min and the peptic peptides trapped and desalted on a 2.1 x 5 mm C18 trap column (Acquity BEH C18 Van-guard pre-column, 1.7 μm, Waters, UK). Trapped peptides were subsequently eluted over 12 min using a 5-36% gradient of acetonitrile in 0.1% v/v formic acid at 40 μl/min. Peptides were separated on a reverse phase column (Acquity UPLC BEH C18 column 1.7 μm, 100 mm x 1 mm (Waters, UK). Peptides were detected on a SYNAPT G2-Si HDMS mass spectrometer (Waters, UK) acquiring over a *m/z* of 300 to 2000, with the standard electrospray ionization (ESI) source and lock mass calibration using [Glu1]-fibrino peptide B (50 fmol/μl). The mass spectrometer was operated at a source temperature of 80°C and a spray voltage of 2.6 kV. Spectra were collected in positive ion mode.

Peptide identification was performed by MS^e^ (Silva et al., 2005) using an identical gradient of increasing acetonitrile in 0.1% v/v formic acid over 12 min. The resulting MS^e^ data were analyzed using Protein Lynx Global Server software (Waters, UK) with an MS tolerance of 5 ppm.

Mass analysis of the peptide centroids was performed using DynamX sotware (Waters, UK). Only peptides with a score >6.4 were considered. The first round of analysis and identification was performed automatically by the DynamX software, however, all peptides (deuterated and non-deuterated) were manually verified at every time point for the correct charge state, presence of overlapping peptides, and correct retention time. Deuterium incorporation was not corrected for back-exchange and represents relative, rather than absolute changes in deuterium levels. Changes in H/D amide exchange in any peptide may be due to a single amide or a number of amides within that peptide. All time points in this study were prepared at the same time and individual time points were acquired on the mass spectrometer on the same day.

#### EMSAs

Electrophoretic mobility shift assays were performed in SEC 50 buffer with 50 nM 5ʹ FAM-labeled precleaved *CYC1* RNA. Complexes were resolved in a 10% native PAGE with 1X TBE at room temperature (100 V for 1.5 hours), and visualized with a Typhoon FLA 7000 (GE Healthcare) using the 473 nm laser and Y520 filter.

#### *In vitro* cleavage and polyadenylation assays

To ensure that the amount of enzyme used in each assay was equivalent between the complexes being compared, we analyzed 10 pmol of the complex to be used in the assay by SDS-PAGE and staining with Instant Blue. We measured the band intensity (integrated density) of Ysh1 (for cleavage assays) or Pap1 (for polyadenylation assays), normalized to the intensity of Cft1 within each respective lane using ImageJ (v. 1.52a). The ratio of normalized band intensities between complexes was used to adjust the calculated concentrations used in the assay.

Dual color cleavage assays were carried out as previously described (Hill et al., 2019) with some modifications. Briefly, reactions were carried out in 1x reaction buffer (5 mM HEPES pH 8.0, 150 mM KOAc, 2 mM MgOAc, 0.05 mM EDTA) supplemented with 3 mM DTT and 1 U/μl RiboLock (Thermo). Reactions were carried out with 100 nM CF IA, 100 nM CF IB, and 50 nM of the indicated CPF complex or variant. Reactions were started by adding 100 nM of the dual-labeled *CYC1* RNA (key resources table).

Polyadenylation assays were carried out as previously described (Casañal et al., 2017; Kumar et al., 2021) under the same conditions as cleavage assays except that 2 mM ATP was added. Reactions were carried out with 100 nM 5ʹ FAM-labeled precleaved *CYC1* RNA (key resources table), 50 nM enzyme, and 100 nM or 450 nM of CF IA and CF IB as indicated.

For each time point in both cleavage and polyadenylation assays, 15 μl of the reaction were stopped with 4 μl of stop solution (130 mM EDTA, 5% SDS, 12 mg/ml proteinase K in 1x reaction buffer) for 5 minutes at 37°C. Samples were mixed with 20 μl of loading buffer (1 M NaCl, 0.1 mg/ml bromophenol blue, 0.1 mg/ml xylene cyanol, 1 mM EDTA, 78% formamide). Reaction products (15 μl of prepared sample) were analyzed on a 10% (polyadenylation assays) or 20% (cleavage assays) urea-PAGE gel.

For both cleavage and polyadenylation assays, gels were scanned in a Typhoon FLA 7000 (GE Healthcare) using the 473 nm laser and Y520 filter for FAM. For cleavage assays, gels were also scanned using the 635 nm laser with the R670 filter for AlexaFluor647. Images were background subtracted (rolling ball radius of 100 pixels with light background) and subject to linear contrast enhancement in ImageJ.

For cleavage reactions, we used ImageJ to measure the integrated intensity of the uncleaved RNA band (FAM channel) at each time point throughout the reaction. For each time point (*t*), we calculated the % of substrate cleaved relative to the initial RNA intensity (*t=0*):

$$\%\;substrate\;cleaved=\left(1-\frac{substrate_{t}}{substrate_{t=0}}\right)\times 100$$

The data was fitted with a global non-linear regression using an exponential plateau function using Prism 8 (v. 8.1.2):

$$Y=Y_{Max}-\left(\;Y_{Max}-Y_{0}\right)e^{\left(-kx\right)}$$

Where Y_Max_ and Y_0_ are globally restrained. The time it took for the enzyme to consume 50% of the maximum substrate consumed by CPF (t_50_) was calculated by solving for x:

$$x=-\left(\frac{ln\left(-\frac{Y-Y_{Max}}{Y_{Max}-Y_{0}}\right)}{k}\right)$$

where *Y* = $\frac{Y_{Max}}{2}$. We used Y_Max_=83.42 (maximum value at plateau, % of initial substrate), Y_0_=0.8474 (starting value, % of initial substrate) and k values specific for each complex.

#### Yeast strains

Yeast strains are listed in the key resources table. To construct JRY101 (Mpe1-mAID) the mAID insert was amplified from pST1933 (Tanaka et al., 2015) using Phusion high-fidelity DNA Polymerase (NEB, cat. no. M0530S) with the Mpe1_F2 and Mpe1_R1 primers, and transformed into YMK728 (WT). For transformation, 100 μl of cells from an overnight culture were harvested, resuspended in transformation buffer (40% PEG 3350, 200 mM lithium acetate, 100 mM DTT, 50 μg salmon sperm DNA (Invitrogen, 15632011)) and heat shocked at 42°C for 30 minutes. Cells were allowed to recover in YEPD for 90 minutes before being plated (Chen et al., 1992). Transformants were selected on YEPD plates supplemented with 500 μg/ml G418 (Sigma, A1720-5G). Colonies were screened by resuspending single colonies in 100 μl of genomic DNA extraction buffer (200 mM LiOAc, 1% SDS) and incubating the suspension at 70°C for 10 minutes. Genomic DNA was ethanol precipitated and recovered by centrifugation. The recovered pellet was resuspended in H_2_O, and cell debris removed by centrifugation. 2 μl of the supernatant containing the genomic DNA (Looke et al., 2011) was used for PCR screening using Mpe1_tag_check_F and KanB_deletion_check primers. Transformants were also tested for auxin sensitivity (0.25 mM–10 mM) in growth curve assays using the Tecan M200 Pro 96-well plate reader with shaking at 30°C. JRY114 was generated by plating 20 μl JRY101 cells grown overnight in rich non-selective media (YEPD) onto synthetic complete media supplemented with 5-FOA (Zymo Research, cat. No. F9001-5) (Boeke et al., 1987). Colonies were verified to be URA3- and G418-resistant. Strains JRY200, JRY208 and JRY210 were generated by transforming JRY101 with pRS314, pRS314-Mpe1 or pRS314-Mpe1^P215G^, respectively.

#### Whole cell extract and immunoblots

Alkaline whole cell extracts were prepared by harvesting 50 ml of cells at OD_600_ 0.5-1 and resuspending cell pellets in 100 μl of 200 mM NaOH. Suspension was incubated at room temperature for 5 minutes, pelleted and resuspended in 50 μl LDS sample buffer (Pierce, 84788) supplemented with 100 mM DTT. Sample was boiled for 3 minutes, pelleted and 5–15 μl of supernatant (whole cell extract) used for immunoblot analysis (Kushnirov, 2000). Whole cell extracts were loaded on 4–12% Bis-Tris SDS-PAGE (Thermo, NP0323BOX) and transferred to nitrocellulose membrane (Whatman Protran BA85, cat. No. 10401196) (25 V, 1 A, 30 min) using Trans Blot Turbo transfer system (Bio-Rad). Membrane was blocked with 5% milk and probed with anti-mAID (1:5,000, MBL Life Science, cat. No. M214-3) or anti-GAPDH-HRP (1:50,000, Invitrogen, cat. No. MA5-15738-HRP). Membranes were visualized with 0.5x ECL (Cytiva, cat. No. RPN2106) using the Gel Doc XR+ system (Bio-Rad).

#### RNA preparation, sequencing, and analysis

##### Auxin-induced depletion of Mpe1

10 ml of WT (YMK728) or degron strains (JRY101, JRY200, JRY208 and JRY210) cells were grown overnight in YEPD + 300 μg/ml G418 at 30°C with 180 rpm shaking. Overnights were subcultured in 60 ml of YEPD to an OD_600_ ∼0.2 and grown to OD_600_ ∼0.9. Cultures were split and treated with either 1 mM auxin (Sigma, cat. No. I3750-100G-A) or an equivalent volume of 100% ethanol (solvent control) for 30 minutes. For YMK728 and JRY101 cells, 5 mM 4-thiouracil (4tU) (Sigma, 440736-1G) was added to each condition during the final 6 minutes of auxin treatment. At the end of treatment, all strains were harvested by centrifugation at 3,000 g for 3 minutes, supernatant discarded and cell pellet flash frozen in liquid N_2_.

##### Spike-in control

*S. pombe* cells for use as a spike-in control were prepared in advance. 10 ml of overnight wild type cells (JU60) were subcultured into 500 ml of YES media and grown to an OD_600_ of ∼0.8. 4tU was added to a final concentration of 5 mM and allowed to incorporate into nascent transcripts for 6 minutes. Cells were harvested and resuspended in PBS to a cell density of 15 OD/ml. 1 ml aliquots were prepared and cell pellets were flash frozen for later use.

##### RNA extraction

Cells from each strain and condition were first resuspended in 0.5 ml TRI reagent each (ThermoFisher, AM9738). One aliquot of frozen *S. pombe* was resuspended in 1 ml TRI reagent and split between the two conditions (*i.e.* 0.5 ml per -/+ auxin) of a given replicate. RNA was chloroform extracted and isopropanol precipitated following TRI reagent manufacturer instructions. Extracted RNA was DNaseI treated (NEB, M0303S) for 1 hour at 37°C followed by phenol-chloroform extraction and isopropanol precipitation. RNA concentration was measured with a Nanodrop. RNA quality was assessed with the Agilent 2100 Bioanalyzer using the RNA 6000 Nano Kit (cat. no. 5067-1511). RNA of good quality (RIN ≥ 7) was used in subsequent analyses.

##### Nascent RNA biotinylation and purification

4tU-labeled RNA was biotinylated and purified as previously described (Dolken et al., 2008) with minor modifications. Briefly, 50 μg of 4tU-labeled RNA (at a final concentration of 50 ng/μl) was biotinylated with 0.2 mg/ml EZ-Link Biotin-HPDP in biotinylation buffer (10 mM HEPES pH 7.5, 1 mM EDTA) at room temperature for 1 hour. Biotinylation reaction was extracted with phenol/chloroform/isoamyl alcohol (125:24:1) followed by isopropanol precipitation. Biotinylated RNA was pulled down with Dynabeads Streptavidin C1 beads and washed twice with high salt wash buffer (100 mM Tris-HCl pH 7.4, 10 mM EDTA, 1 M NaCl, 0.1 % Tween-20). Biotinylated RNA was eluted twice from beads with 100 μl freshly prepared 5% β-mercaptoethanol. Eluted RNA was ethanol precipitated with GlycoBlue (Invitrogen, AM9515) following the manufacturer instructions. The precipitated RNA was resuspended in 30 μl H_2_O, and the quality assessed using a Nanodrop and Bioanalyzer as described above.

##### First strand cDNA and qPCR

First strand cDNA synthesis was performed using SuperScript III Reverse Transcriptase (Invitrogen, 18080-093) following product instructions using 1 μg of total or nascent RNA and 2.5 μM oligo(dT)/random hexamer oligo mix. 10 μl qPCR reactions (using 1:10 diluted cDNA) were assembled using Power SYBR Green PCR master mix (Thermo, cat. no. 4367659) following product instructions and primers listed in Table S1. qPCRs were carried out in a 384-well Agilent ViiA 7 instrument. All reactions were performed in technical triplicates and Ct values averaged. To normalize to *S. pombe* spike-in controls we first averaged the Ct values of three *S. pombe* transcripts (*S. pombe* Ct_k_: act-1, adh-1 and gpd-3) to obtain a single spike-in value for each condition and replicate (Ct_spike-in_):

$$Ct_{spike-in}=\frac{\sum_{n=1}^{k}S.pombe\;Ct_{k}}{k}$$

where *k* is the number of *S. pombe* transcripts (k=3).

We next normalized each Ct value from experimental *S. cerevisiae* samples to its corresponding Ct_spike-in_ value:

$$\Delta Ct=Ct_{i}-Ct_{spike-in}$$

where *i* is individual replicates for each condition (*i.e.* total, nascent, -auxin, +auxin). Next we calculated the difference between +auxin and –auxin conditions, and calculated the fold change:

$$\Delta \Delta Ct=\Delta Ct_{+auxin}-\Delta Ct_{-auxin}$$

$$Fold\;change=2^{-\Delta \Delta Ct}$$

To determine the level of readthrough for a given gene (Figure S7B), we normalized the fold change of the downstream position to the fold change of the genic region 1 kb upstream.

##### Library preparation and sequencing

Ribosomal RNA was depleted using the NEBNext rRNA depletion kit (NEB) following the product instructions. Depletion was confirmed with the Agilent 2100 Bioanalyzer using the RNA 6000 Nano kit.

Total and nascent RNA libraries were prepared using the NEBNext Ultra II Directional RNA Library Prep Kit for Illumina (NEB, E7760S) following the rRNA depleted RNA protocol. Libraries were pooled and single-end sequenced (50 bp) in a HiSeq 4000 instrument (Cancer Research UK).

##### Sequencing data processing and analysis

Data was trimmed with TrimGalore 0.4.5 (-q 20) and aligned to merged reference genomes of *S. cerevisiae* (R64-1-1) and *S. pombe* (ASM294v2) using STAR (v. 2.6.0a) (Dobin et al., 2013). Aligned reads were filtered using samtools view with the following flags: -b –q 7 -F 1284. Strand-specific read quantification per genomic feature was carried with the featureCounts program of Rsubread (v. 2.0.1) (Liao et al., 2014), using the filtered reads and the merged genomic features of *S. cerevisiae* and *S. pombe*. The output of featureCounts was used with the RUVg method of RUV-seq (v. 1.20.0) (Risso et al., 2014) which used the mapped reads from *S. pombe* spike-ins to remove unwanted variability in the data. This spike-in corrected data was then used in DESeq2 (v. 1.26.0) (Love et al., 2014) (lfcThreshold = 0, alpha = 0.05, pAdjustMethod = "fdr") to identify differentially expressed genes in the *S. cerevisiae* data.

For visualization of the data in Integrated Genome Viewer (v. 2.4.11) (Robinson et al., 2011), filtered reads from replicates were merged using samtools merge –c –r –f followed by splitting strand-specific aligned reads with samtools view –b –F 20 for the plus strand, and –b –f 16 for the minus strand. Strand-split data was then sorted (samtools sort) and indexed (samtools index –b). To visualize the data we used deeptools bamCoverage (v. 3.1.3) (Ramirez et al., 2016) to generate binned data across the genome (--normalizeUsing CPM --binSize 10 --smoothLength 20). To generate the scatter plots in Figure 6C, the output of bamCoverage was used in deeptools multiBigWigSummary bins (with --binSize 1000) or BED-file (with the ORF-T annotation (Xu et al., 2009)). Scatter plots were generated in R (v. 3.6.0). SeqPlots was used for k-means clustering (k=4; k value determined empirically to give clusters with distinct patterns) of the strand-specific nascent RNA-seq data within a 1 kb window centered around the polyadenylation site. SeqPlots (Stempor and Ahringer, 2016) was also used to generate the metagene plots in Figure 6B. The log_2_ fold change for each gene in Figure 6E was calculated with DESeq2 and boxplots generated in R.

### Quantification and statistical analysis

Statistical analyses were performed using Prism (Figures 5C, S6A, S6D, S7A, and S7B), R (Figures 6C, 6E, and S6E–S6G) and SeqPlots (Figure 6B). Details on number of replicates or number of genes, error estimates, goodness of model fit (R^2^), statistical tests and significance cutoff can be found in the respective figure legends.

## Data Availability

- The models and maps for the structures presented have been deposited in the PDB and EMDB. Sequencing data have been deposited in ArrayExpress. The accession numbers are listed in the key resources table. Original gel files have been deposited on Mendeley Data: https://dx.doi.org/10.17632. These are all publicly available as of the date of publication. Micrographs reported in this paper will be shared by the lead contact upon request. - This paper does not report original code. - Any additional information required to reanalyze the data reported in this paper is available from the lead contact upon request.

## Supporting citations

The following references appear in the supplemental information: Chen et al. (2013); Thompson et al. (1997).

## Supplemental information

Document S1. Figures S1–S7 and Table S1

Data S1. Crosslinked residue pairs for crosslinking mass spectrometry analysis of polymerase module-Mpe1-Cft2(S)-RNA, related to Figure 4

Document S2. Article plus supplemental information
