# Supplementary material for: Mpe1 senses the binding of pre-mRNA and controls 3′ end processing by CPF
Source: Mol Cell. 2022 Jul 7;82(13):2490–2504.e12. doi: 10.1016/j.molcel.2022.04.021 (PMC9380774; doi:10.1016/j.molcel.2022.04.021)
Supplement: Document S1. Figures S1–S7 and Table S1 [file mmc1.pdf]

Molecular Cell, Volume *82*

Supplemental information

Mpe1 senses the binding of pre-mRNA and controls 3′ end processing by CPF

Juan B. Rodríguez-Molina, Francis J. O'Reilly, Holly Fagarasan, Eleanor Sheekey, Sarah Maslen, J. Mark Skehel, Juri Rappsilber, and Lori A. Passmore

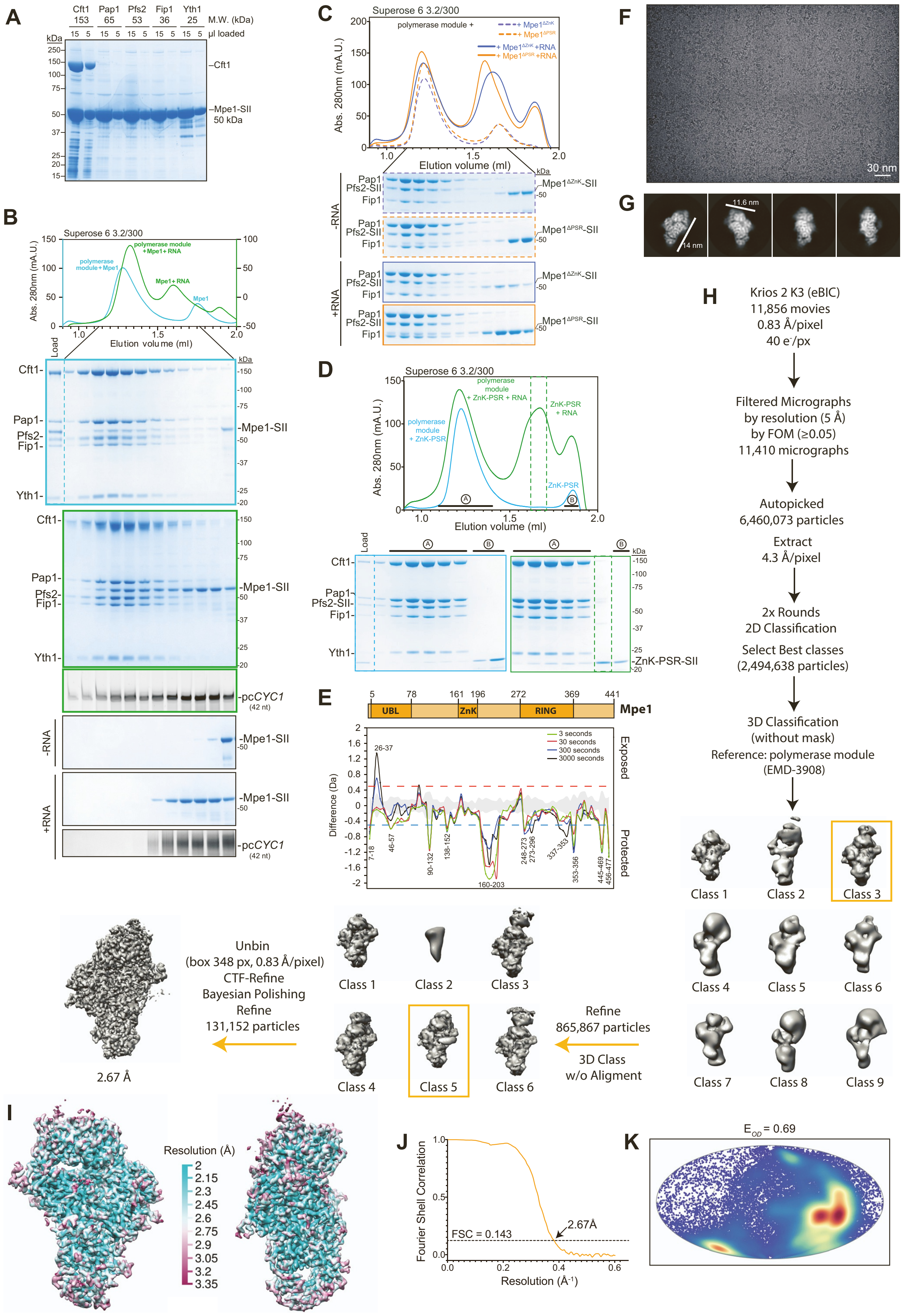
A
Cft1 153
Pap1 65
Pfs2 53
Fip1 36
Yth1 25
M.W. (kDa)
µl loaded
kDa
Cft1
Mpe1-SII 50 kDa
B
Superose 6 3.2/300
Abs. 280nm (mA.U.)
polymerase module +Mpe1+ RNA
polymerase module+Mpe1
Mpe1+ RNA
Mpe1
Elution volume (ml)
Load
Cft1-
Pap1-
Pfs2-
Fip1-
Yth1-
Mpe1-SII
pcCYC1 (42 nt)
-RNA
+RNA
C
Superose 6 3.2/300
polymerase module +
+ Mpe1ΔZnK
+ Mpe1ΔPSR
+ Mpe1ΔZnK +RNA
+ Mpe1ΔPSR +RNA
Abs. 280nm (mA.U.)
Elution volume (ml)
Pap1
Pfs2-SII
Fip1
Mpe1ΔZnK-SII
Mpe1ΔPSR-SII
-RNA
+RNA
D
Superose 6 3.2/300
polymerase module + ZnK-PSR + RNA
ZnK-PSR + RNA
polymerase module + ZnK-PSR
ZnK-PSR
Abs. 280nm (mA.U.)
Elution volume (ml)
Load
Cft1-
Pap1-
Pfs2-SII-
Fip1-
Yth1-
ZnK-PSR-SII
E
UBL
ZnK
RING
Mpe1
3 seconds
30 seconds
300 seconds
3000 seconds
Difference (Da)
Exposed
Protected
F
30 nm
G
14 nm
11.6 nm
H
Krios 2 K3 (eBIC)
11,856 movies
0.83 Å/pixel
40 e-/px
Filtered Micrographs
by resolution (5 Å)
by FOM (≥0.05)
11,410 micrographs
Autopicked
6,460,073 particles
Extract
4.3 Å/pixel
2x Rounds
2D Classification
Select Best classes
(2,494,638 particles)
3D Classification
(without mask)
Reference: polymerase module
(EMD-3908)
Class 1
Class 2
Class 3
Class 4
Class 5
Class 6
Class 7
Class 8
Class 9
Refine
865,867 particles
3D Class
w/o Aligment
Unbin
(box 348 px, 0.83 Å/pixel)
CTF-Refine
Bayesian Polishing
Refine
131,152 particles
2.67 Å
I
Resolution (Å)
J
Fourier Shell Correlation
FSC = 0.143
2.67Å
Resolution (Å-1)
K
EOD = 0.69

Figure S1. Mpe1 interacts with the polymerase module. Related to Figure 1

(A) SDS-PAGE of pulldowns from insect cell lysates co-expressing StrepII-tagged (SII) Mpe1 (bait) with individual subunits of the polymerase module. For each pulldown, two different amounts of eluate were loaded as indicated. Expected molecular weights of each subunit are indicated.

(B) Co-purification of Mpe1 with the polymerase module. Size exclusion chromatography was performed for polymerase module mixed with Mpe1-SII without (blue) or with (green) a 5' FAM-labeled precleaved *CYC1* RNA (pc*CYC1*). Overlaid chromatograms are shown in the top panel. Fractions were analyzed by SDS-PAGE for protein and by denaturing urea-PAGE for RNA. Borders of gels correspond to colors of chromatogram traces. A sample of the injected input is shown in the lane labeled 'Load'. The three panels at the bottom show SDS-PAGE and urea-PAGE gels of fractions of Mpe1 alone (-RNA) or in complex with precleaved *CYC1* RNA (+RNA) generated by *in vitro* transcription. Free Mpe1 co-elutes with RNA confirming a direct association of Mpe1 with RNA.

(C) Analytical size exclusion chromatography of polymerase module with Mpe1$^{\Delta ZnK}$ or Mpe1$^{\Delta PSR}$, with or without *in vitro* transcribed precleaved *CYC1* RNA. Outlines of gels match the chromatograms on the top panel.

(D) Analytical size exclusion chromatography of polymerase module with the ZnK-PSR of Mpe1, with or without *in vitro* transcribed precleaved *CYC1* RNA. Outlines of gels match chromatogram colors. The fractions analyzed by SDS-PAGE are indicated. A fraction of the ZnK-PSR in complex with RNA is indicated by a green rectangle with dashed-lines. A sample of the injected input is shown in the lane labeled 'Load'.

(E) Hydrogen-deuterium exchange mass spectrometry difference plot (Mpe1-polymerase module versus Mpe1) showing peptides of Mpe1 that are protected (negative) and exposed (positive) by interaction with the polymerase module. A domain diagram of Mpe1 is shown above. Several regions of Mpe1 are protected including the zinc knuckle, the RING finger and a C-terminal region.

(F) Representative cryo-EM micrograph of polymerase module-Mpe1-RNA complex.

(G) 2D class averages of the polymerase module-Mpe1-RNA complex.

(H) Schematic of data collection, processing and map reconstruction strategy for the polymerase module-Mpe1-RNA complex (see STAR Methods for details). The nominal resolution of the final map is shown. Maps were flipped on the Z-axis following final refinement to match correct stereochemistry before model building.

(I) Local resolution distribution of the polymerase module-Mpe1-RNA map.

(J) Fourier shell correlation (FSC) plot of sharpened cryo-EM map of the polymerase module-Mpe1-RNA complex.

(K) Mollweide projection of orientation distribution of particles in the final cryo-EM map of polymerase module-Mpe1-RNA complex. Efficiency of orientation distribution (E_{OD}) is 0.69, which indicates a minor Fourier space gap (Naydenova et al., 2017).

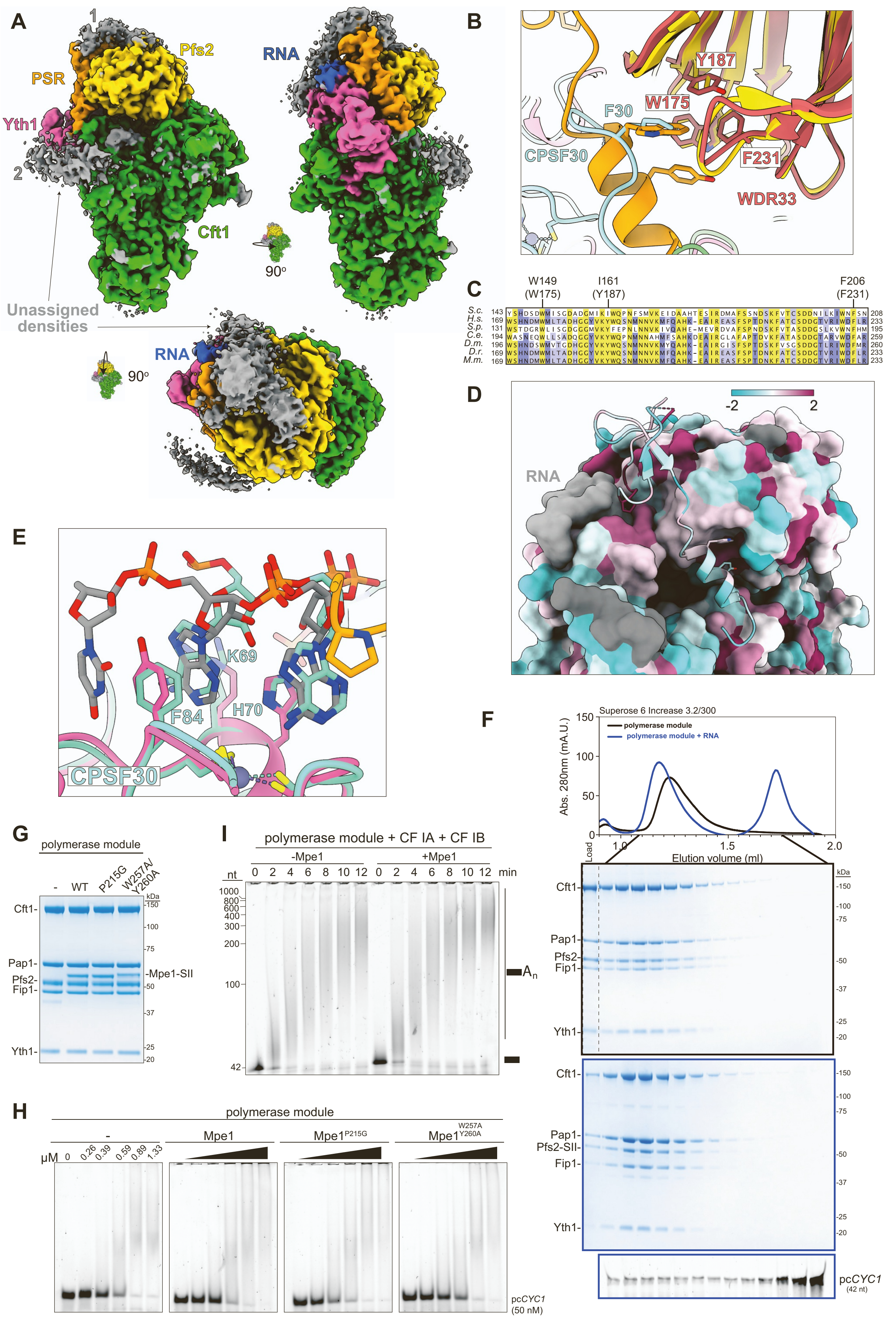

A
1
Pfs2
PSR
Yth1
2
Cft1
RNA
90°
Unassigned densities
B
Y187
W175
F30
CPSF30
F231
WDR33
C
W149 (W175)
I161 (Y187)
F206 (F231)
S.c. 143
H.s. 169
S.p. 131
C.e. 194
D.m. 196
D.r. 169
M.m. 169
208
233
195
259
260
233
233
D
-2
2
RNA
E
K69
F84
H70
CPSF30
F
Superose 6 Increase 3.2/300
polymerase module
polymerase module + RNA
Abs. 280nm (mA.U.)
150
100
50
0
Load
1.0
1.5
2.0
Elution volume (ml)
kDa
Cft1-
Pap1-
Pfs2-
Fip1-
Yth1-
Pfs2-SII-
150
100
75
50
37
25
20
pcCYC1
(42 nt)
G
polymerase module
-
WT
P215G
W257A/ Y260A
Mpe1-SII
H
polymerase module
Mpe1
Mpe1 P215G
Mpe1 W257A Y260A
µM
0
0.26
0.39
0.59
0.89
1.33
pcCYC1
(50 nM)
I
polymerase module + CF IA + CF IB
-Mpe1
+Mpe1
nt
0 2 4 6 8 10 12 0 2 4 6 8 10 12 min
1000
800
600
400
300
200
100
42
An

Figure S2. Mpe1 senses RNA binding by the polymerase module. Related to Figures 1 and 2.

(A) Cryo-EM map of polymerase module-Mpe1-RNA rendered at a lower contour level to show additional lower-resolution densities (grey) that could not be modeled. Additional density 1 is positioned over a positively-charged patch on Pfs2, but since it is poorly ordered, we were not able to confirm its identity. Additional density 2 in front of the Cft1 C-terminal domain may represent an additional, poorly-resolved interaction between Mpe1 and Cft1. Additional unassigned densities within Cft1 (also in grey) likely correspond to flexible loops and could not be confidently modeled at higher contour levels.

(B) Structure alignment of mPSF (PDB: 6fuw), and polymerase module-Mpe1-RNA (this study). Yeast Mpe1 (orange) and human CPSF30 (cyan) insert into the equivalent hydrophobic pocket of Pfs2/WDR33 (yellow/salmon) via a bulky hydrophobic residue (W257 in Mpe1, F30 in CPSF30).

(C) Multiple sequence alignment of Pfs2 orthologues across model eukaryotes. Residues highlighted in yellow are conserved across most eukaryotes; those in purple are partially conserved. See Figure 1F for species abbreviations.

(D) Surface rendering of the polymerase module-Mpe1-RNA model, except Mpe1 which is in cartoon representation, colored by the entropy-based conservation index implemented by AL2CO in ChimeraX. Pfs2 shows high conservation in the PSR helix binding site (e.g. W149/W175, I161/Y187 and F206/F231 in yeast/humans respectively) and near the N-terminal region of the PSR. Overall, the Mpe1 PSR buries a combined surface area of ~1,200 $Å^2$ on the polymerase module, a relatively modest area that is consistent with the small number of residues (Chen et al., 2013) that stabilize the interaction between the PSR and the polymerase module.

(E) Structure alignment of RNA-bound zinc finger 2 of CPSF30 of mPSF (green; PDB: 6fbs), and Yth1 of the polymerase module-Mpe1-RNA structure (pink and grey; this study). The recognition mechanism of A_1A_2 in the PAS is conserved.

(F) Size exclusion chromatogram of polymerase module (black) (top) or polymerase module in complex with 5' FAM-labeled precleaved *CYC1* RNA (pc*CYC1*) (blue) (bottom). Protein and RNA from the indicated fractions were analyzed on SDS-PAGE and urea-PAGE (bottom panels). The outlines of the gels correspond to the color of the chromatograms. The polymerase module-RNA complex elutes earlier than the polymerase module alone, consistent with complex formation. A sample of the injected input is shown in the lane labeled ‘Load’.

(G) SDS-PAGE of 10 pmol of polymerase module alone (-) or in complex with Mpe1 (WT), $Mpe1^{P215G}$ or $Mpe1^{W257A/Y260A}$.

(H) Representative electrophoretic mobility shift assays (EMSAs) of precleaved *CYC1* RNA (pc*CYC1*) with polymerase module alone or in complex with Mpe1 (WT), $Mpe1^{P215G}$ or $Mpe1^{W257A/Y260A}$ as indicated. Concentrations of protein used are shown for the first gel (left) and are the same for Mpe1-containing complexes. N = 4.

(I) Polyadenylation assays similar to Figure 2E, except that 100 nM CF IA and CF IB were included in the reaction. CF IA and CF IB stimulate polyadenylation activity to comparable levels in both complexes.

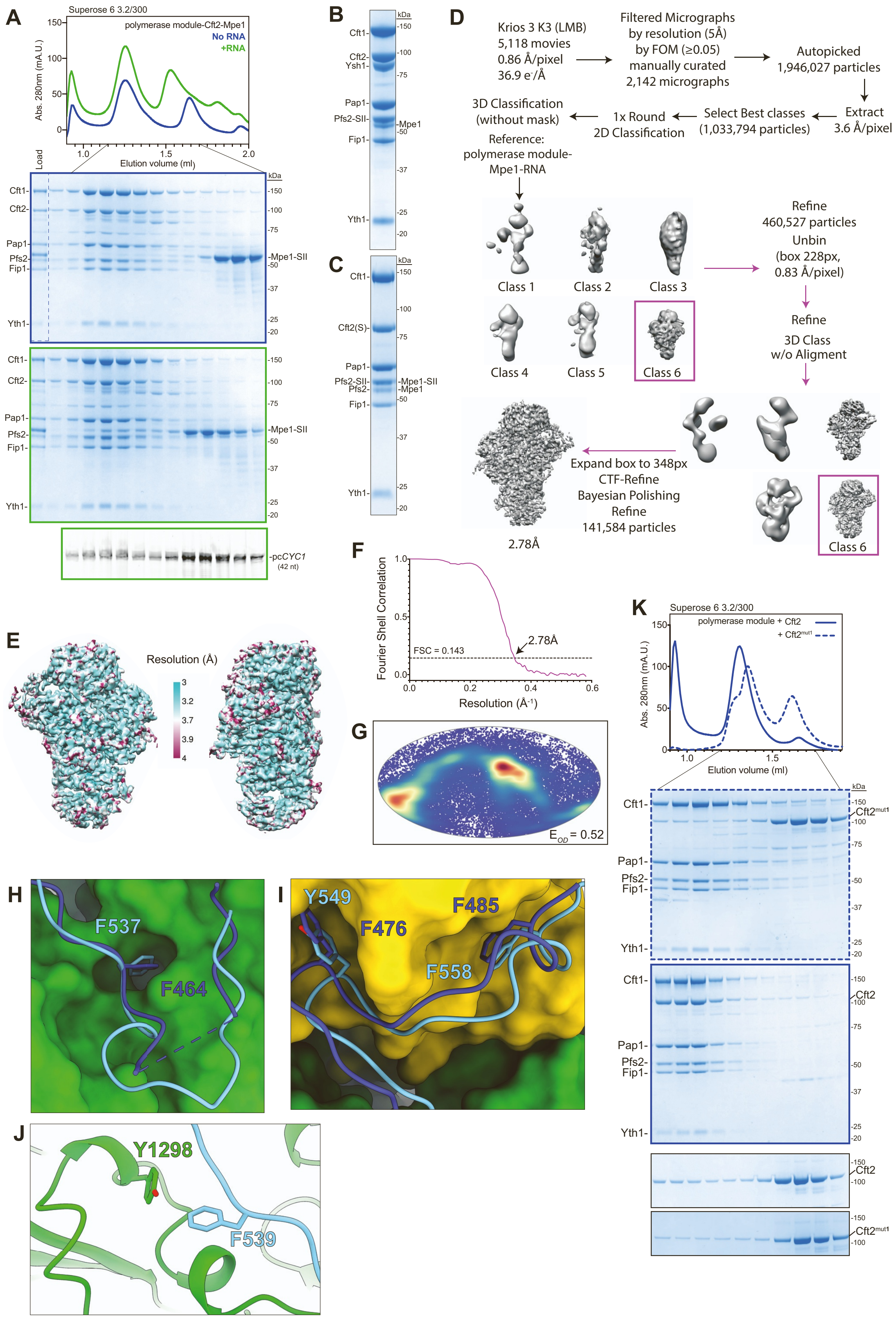

A
Superose 6 3.2/300
polymerase module-Cft2-Mpe1
No RNA
+RNA
Abs. 280nm (mA.U.)
Elution volume (ml)
Load
Cft1-
Cft2-
Pap1-
Pfs2-
Fip1-
Yth1-
-Mpe1-SII
kDa
-pcCYC1
(42 nt)
B
Cft1-
Cft2-
Ysh1-
Pap1-
Pfs2-SII-
Fip1-
Yth1-
-Mpe1
C
Cft1-
Cft2(S)-
Pap1-
Pfs2-SII-
Pfs2-
Fip1-
Yth1-
-Mpe1-SII
-Mpe1
D
Krios 3 K3 (LMB)
5,118 movies
0.86 Å/pixel
36.9 e-/Å
Filtered Micrographs
by resolution (5Å)
by FOM (≥0.05)
manually curated
2,142 micrographs
Autopicked
1,946,027 particles
Extract
3.6 Å/pixel
Select Best classes
(1,033,794 particles)
1x Round
2D Classification
3D Classification
(without mask)
Reference:
polymerase module-
Mpe1-RNA
Class 1
Class 2
Class 3
Class 4
Class 5
Class 6
Refine
460,527 particles
Unbin
(box 228px,
0.83 Å/pixel)
Refine
3D Class
w/o Aligment
Expand box to 348px
CTF-Refine
Bayesian Polishing
Refine
141,584 particles
2.78Å
Class 6
E
Resolution (Å)
3
3.2
3.7
3.9
4
F
Fourier Shell Correlation
FSC = 0.143
2.78Å
Resolution (Å-1)
G
EOD = 0.52
H
F537
F464
I
Y549
F476
F485
F558
J
Y1298
F539
K
Superose 6 3.2/300
polymerase module + Cft2
+ Cft2mut1
Abs. 280nm (mA.U.)
Elution volume (ml)
Cft1-
Pap1-
Pfs2-
Fip1-
Yth1-
Cft2mut1
Cft2
kDa

Figure S3. Cryo-EM of polymerase module-Cft2(S)-Mpe1-RNA. Related to Figure 3

(A) Corresponding uncropped gels for Figure 3A. A sample of the injected input is shown in the lane labeled 'Load'.

(B) Representative Coomassie-stained SDS-PAGE of the combined nuclease and polymerase modules, termed CPF_{core} complex (Hill et al., 2019).

(C) Representative Coomassie-stained SDS-PAGE of the polymerase module-Mpe1-Cft2(S)-RNA complex used for cryo-EM sample preparation. Complex was purified by size exclusion chromatography as described for the complex with full length Cft2. Carryover 3C protease from previous purification steps of Cft2(S) led to partial cleavage of the SII tags on Mpe1 and Pfs2. The shorter version of Cft2 (Cft2(S)) was used because the C-terminal region causes problems with particle distribution in ice.

(D) Schematic of data collection, processing and 3D reconstruction of polymerase module-Cft2(S)-Mpe1-RNA complex. The maps contained density only for polymerase module and Cft2(S). Maps were flipped on the Z-axis following final refinement to match correct stereochemistry before model building.

(E) Local resolution distribution of unsharpened cryo-EM map of the polymerase module-Cft2(S) reconstruction.

(F) Fourier shell correlation plot of sharpened cryo-EM map of the polymerase module-Cft2(S) complex. Nominal resolution was determined at FSC = 0.143 threshold.

(G) Mollweide projection of orientation distribution of particles in the final cryo-EM map of the polymerase module-Cft2(S) complex. Efficiency of orientation distribution (E_{OD} = 0.52) indicates a modest Fourier space gap (Naydenova et al., 2017).

(H-I) Alignment of polymerase module-Cft2(S) structure with the mPSF-PIM structure (PDB: 6urg). The most conserved residues of the yPIM (light blue) and PIM (purple) are shown. Cft1 (green) and Pfs2 (yellow) are shown in surface representation.

(J) A loop (A1294-S1303) of Cft1 (green) that is disordered in the absence of Cft2 is stabilized upon binding to the yPIM of Cft2 (light blue).

(K) Analytical size exclusion chromatography of polymerase module in complex with a full-length mutant of Cft2 ($Cft2^{mut1}$, dashed blue gel outline and chromatogram). $Cft2^{mut1}$ carries three point mutations in conserved residues that make direct contacts with the polymerase module (F537A, Y549A, F558A). Polymerase module in complex with full-length WT Cft2 was also analyzed (Cft2, solid blue gel outline and chromatogram). Bottom panels show the elution pattern of Cft2 or $Cft2^{mut1}$ alone.

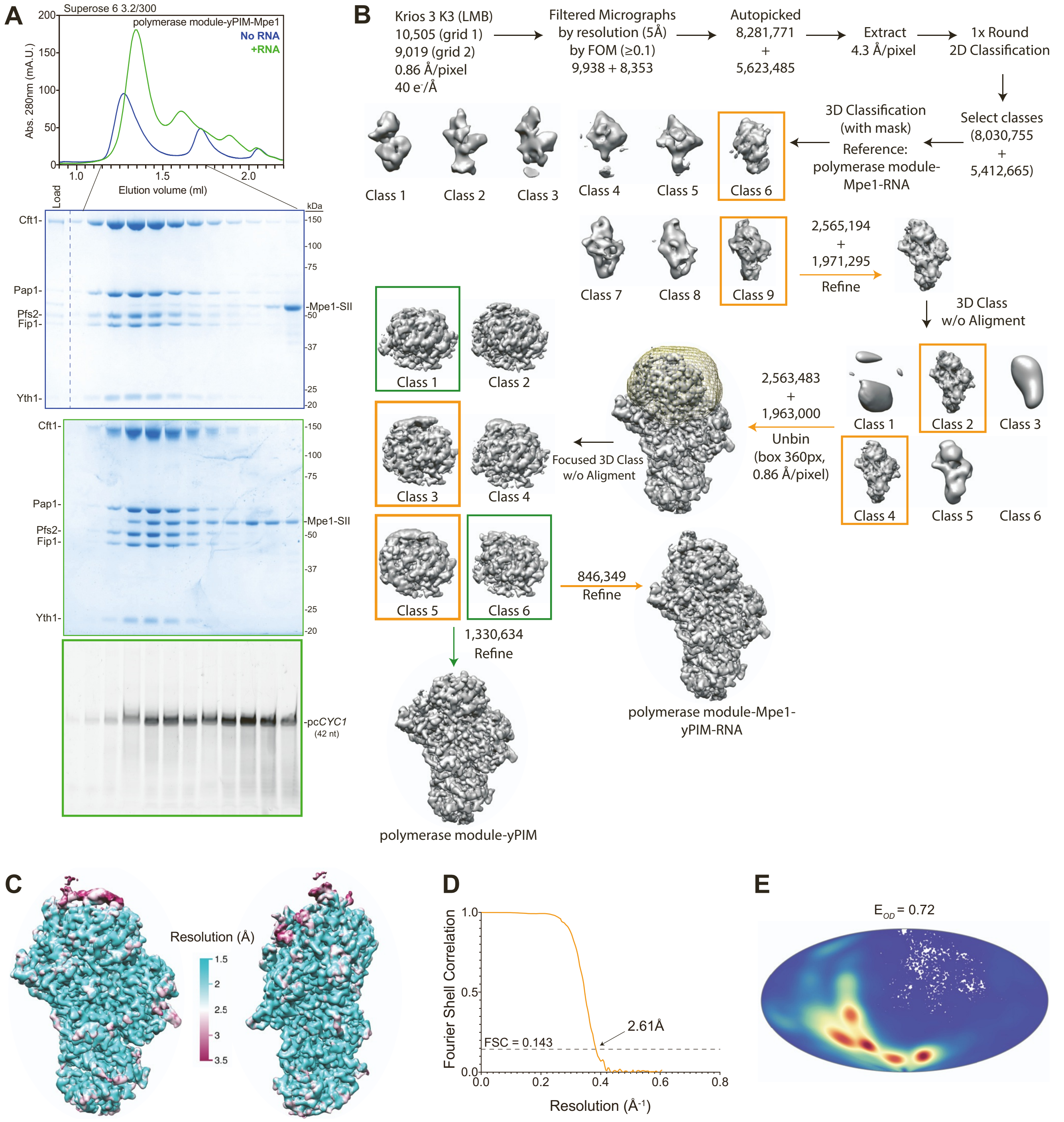

Figure S4. Cryo-EM of the polymerase module-yPIM-Mpe1-RNA complex. Related to Figure 4.
(A) Size exclusion chromatography performed with the polymerase module in complex with the yPIM of Cft2 and Mpe1 either without (blue) or with (green) precleaved *CYC1* RNA (pc*CYC1*). The chromatogram (top), Coomassie-stained SDS-PAGE of indicated fractions (middle two panels) and urea-PAGE gel of 5' FAM-labeled RNA from the indicated fractions (bottom panel) are shown. Outline color of gels correspond to the colors of the chromatogram profiles. A sample of the injected input is shown in the lane labeled 'Load'.
(B) Schematic of cryo-EM data collection and processing for the polymerase module-Mpe1-yPIM-RNA complex. Data were collected from two grids, and the particle number processed from each is indicated separately until merging. Focused classification with a mask around the top region of the map (yellow mesh) was necessary to parse through particles that contained or lacked clear density for the Mpe1 PSR. Selected classes are indicated with orange (with Mpe1 PSR density) or green (without Mpe1 PSR density) boxes.

(C) Cryo-EM map of polymerase module-Mpe1-yPIM-RNA complex colored by local resolution.
(D) Fourier shell correlation plot of sharpened cryo-EM map of the polymerase module-Mpe1-yPIM-RNA complex. Nominal resolution was determined at FSC = 0.143 threshold.
(E) Mollweide projection of orientation distribution of particles in the final polymerase module-Mpe1-yPIM-RNA cryo-EM map. Efficiency of orientation distribution (E_{OD} = 0.72) indicates minor Fourier space gap.

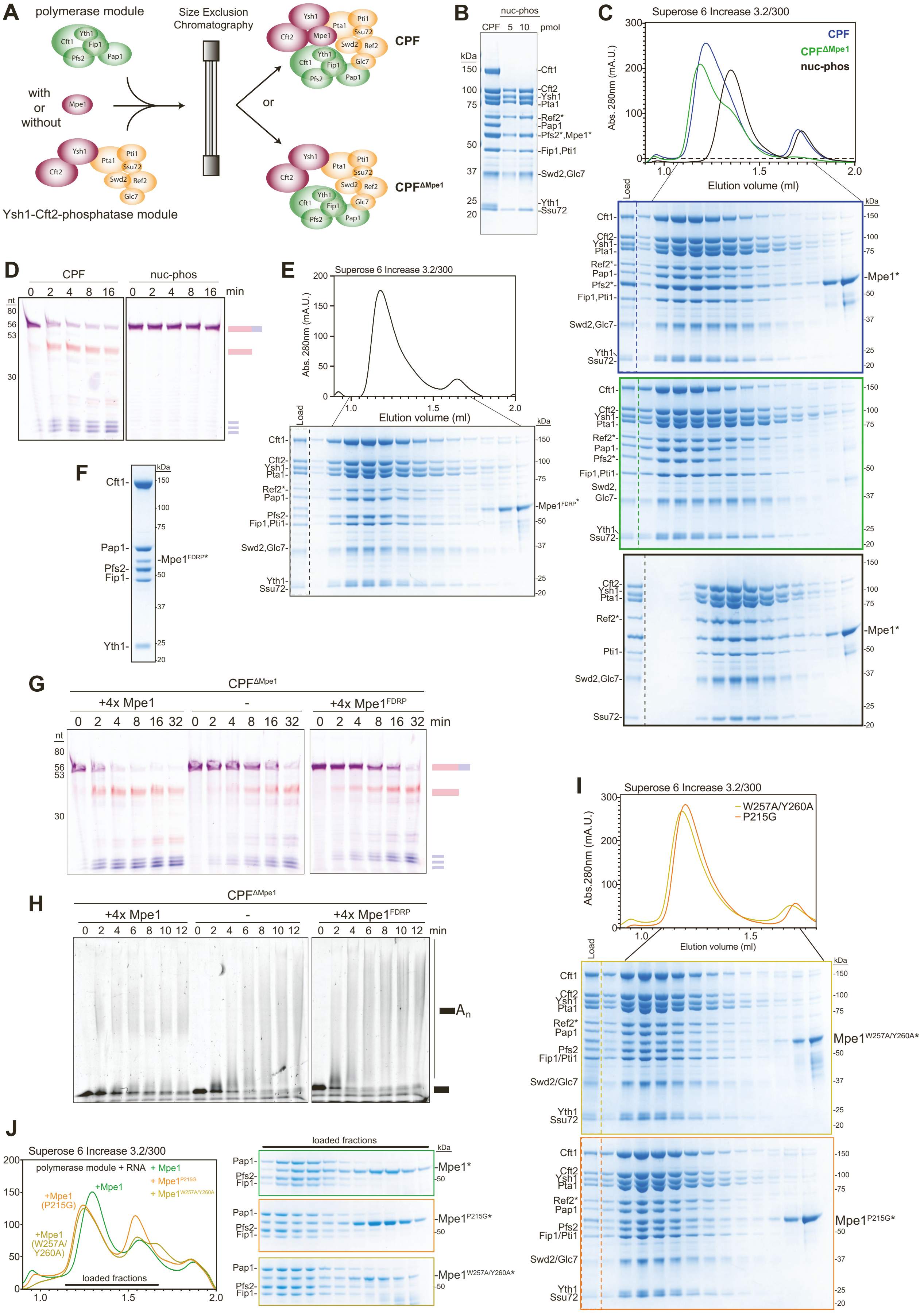

A
polymerase module
Cft1
Yth1
Fip1
Pfs2
Pap1
with or without
Mpe1
Ysh1
Cft2
Pta1
Pti1
Ssu72
Swd2
Ref2
Glc7
Ysh1-Cft2-phosphatase module
Size Exclusion Chromatography
or
CPF
CPF$^{\Delta Mpe1}$
B
nuc-phos
CPF 5 10 pmol
kDa
150 100 75 50 37 25 20
-Cft1
-Cft2
-Ysh1
-Pta1
-Ref2*
-Pap1
-Pfs2*,Mpe1*
-Fip1,Pti1
-Swd2,Glc7
-Yth1
-Ssu72
C
Superose 6 Increase 3.2/300
CPF
CPF$^{\Delta Mpe1}$
nuc-phos
Abs. 280nm (mA.U.)
Elution volume (ml)
Load
kDa
-Mpe1*
D
CPF
nuc-phos
0 2 4 8 16 min
nt
80 56 53 30
E
Superose 6 Increase 3.2/300
Abs. 280nm (mA.U.)
Elution volume (ml)
-Mpe1^{FDRP}*
F
Cft1-
Pap1-
Pfs2-
Fip1-
Yth1-
-Mpe1^{FDRP}*
G
CPF$^{\Delta Mpe1}$
+4x Mpe1
-
+4x Mpe1^{FDRP}
0 2 4 8 16 32 min
H
CPF$^{\Delta Mpe1}$
+4x Mpe1
-
+4x Mpe1^{FDRP}
0 2 4 6 8 10 12 min
A_n
I
Superose 6 Increase 3.2/300
W257A/Y260A
P215G
Abs.280nm (mA.U.)
Elution volume (ml)
Mpe1$^{W257A/Y260A}$*
Mpe1^{P215G}*
J
Superose 6 Increase 3.2/300
polymerase module + RNA
+ Mpe1
+ Mpe1^{P215G}
+ Mpe1$^{W257A/Y260A}$
+Mpe1
+Mpe1 (P215G)
+Mpe1 (W257A/ Y260A)
loaded fractions
Pap1-
Pfs2-
Fip1-
-Mpe1*
-Mpe1^{P215G}*
-Mpe1$^{W257A/Y260A}$*

Figure S5. Purification and assay of recombinant CPF carrying variants of Mpe1. Related to Figure 5.
(A) Schematic of CPF reconstitution and purification via size exclusion chromatography. Purified polymerase module was combined with a complex carrying all the subunits of the phosphatase module (Pta1, Pti1, Ssu72, Ref2-SII, Swd2 and Glc7) and two subunits of the nuclease module (Ctf2 and Ysh1). The assembled complex was then purified by size exclusion chromatography. The same procedure allowed us to reconstitute full CPF including Mpe1.
(B) SDS-PAGE of 10 pmol CPF, and 5 or 10 pmol of the combined nuclease and phosphatase modules (nuc-phos) complex as indicated. Subunits with an asterisk (*) are SII-tagged.
(C) Size exclusion chromatogram of reconstituted CPF (blue), CPF$^{\Delta Mpe1}$ (green) or CPF lacking the polymerase module (nuc-phos, black) is shown in the top panel. SDS-PAGE of indicated fractions for each of the reconstituted complexes are shown below. Border colors of gels correspond to chromatogram trace colors. Subunits with an asterisk (*) are SII-tagged. A sample of the injected input is shown in the lane labeled 'Load'.
(D) Representative urea-PAGE of a dual-color *in vitro* cleavage assay using a 5' FAM (red) and 3' Alexa647 (blue)-labeled uncleaved *CYC1* RNA substrate with reconstituted CPF or CPF lacking the polymerase module (nuc-phos).
(E) Size exclusion chromatogram (top) and corresponding SDS-PAGE (bottom) of CPF with an Mpe1 UBL mutant (Mpe1^{FDRP}; F9A, D45K, R76E, P78G). Mpe1^{FDRP} did not stably integrate into CPF. Subunits with an asterisk (*) are SII-tagged. A sample of the injected input is shown in the lane labeled 'Load'.
(F) Representative SDS-PAGE of polymerase module in complex Mpe1^{FDRP} after purification by size exclusion chromatography.
(G) *In vitro* dual-color cleavage assays using CPF$^{\Delta Mpe1}$. WT Mpe1 or Mpe1^{FDRP} were added to the reaction in *trans* as indicated (4x the concentration of CPF$^{\Delta Mpe1}$ in the reaction corresponds to 200 nM Mpe1).
(H) *In vitro* polyadenylation assays using 5' FAM-labeled precleaved *CYC1* substrate and CPF$^{\Delta Mpe1}$. WT or Mpe1^{FDRP} were added to the reaction in *trans* as indicated (4x the concentration of CPF$^{\Delta Mpe1}$ in the reaction corresponds to 200 nM Mpe1). Note the difference in poly(A) tail length distribution throughout the time courses. Specifically, the rate of polyadenylation with Mpe1^{FDRP} is reduced compared to WT Mpe1, but increased compared to no Mpe1, particularly in the first time points.
(I) Size exclusion chromatogram (top) and SDS-PAGE of indicated fractions (bottom) of CPF assemblies using purified Mpe1$^{W257A/Y260A}$ or Mpe1^{P215G}. Border colors of gels correspond to chromatogram trace colors. Both Mpe1 variants incorporate into and co-elute with CPF. Subunits with an asterisk (*) are SII-tagged. A sample of the injected input is shown in the lane labeled 'Load'.
(J) Analytical size exclusion chromatography of polymerase module in complex with precleaved *CYC1* RNA and Mpe1 (green), Mpe1^{P215G} (orange) or Mpe1$^{W257A/Y260A}$ (brown). A black line below the chromatogram traces (left) indicates fractions analyzed by SDS-PAGE (right). Chromatogram colors correspond to gel outlines.

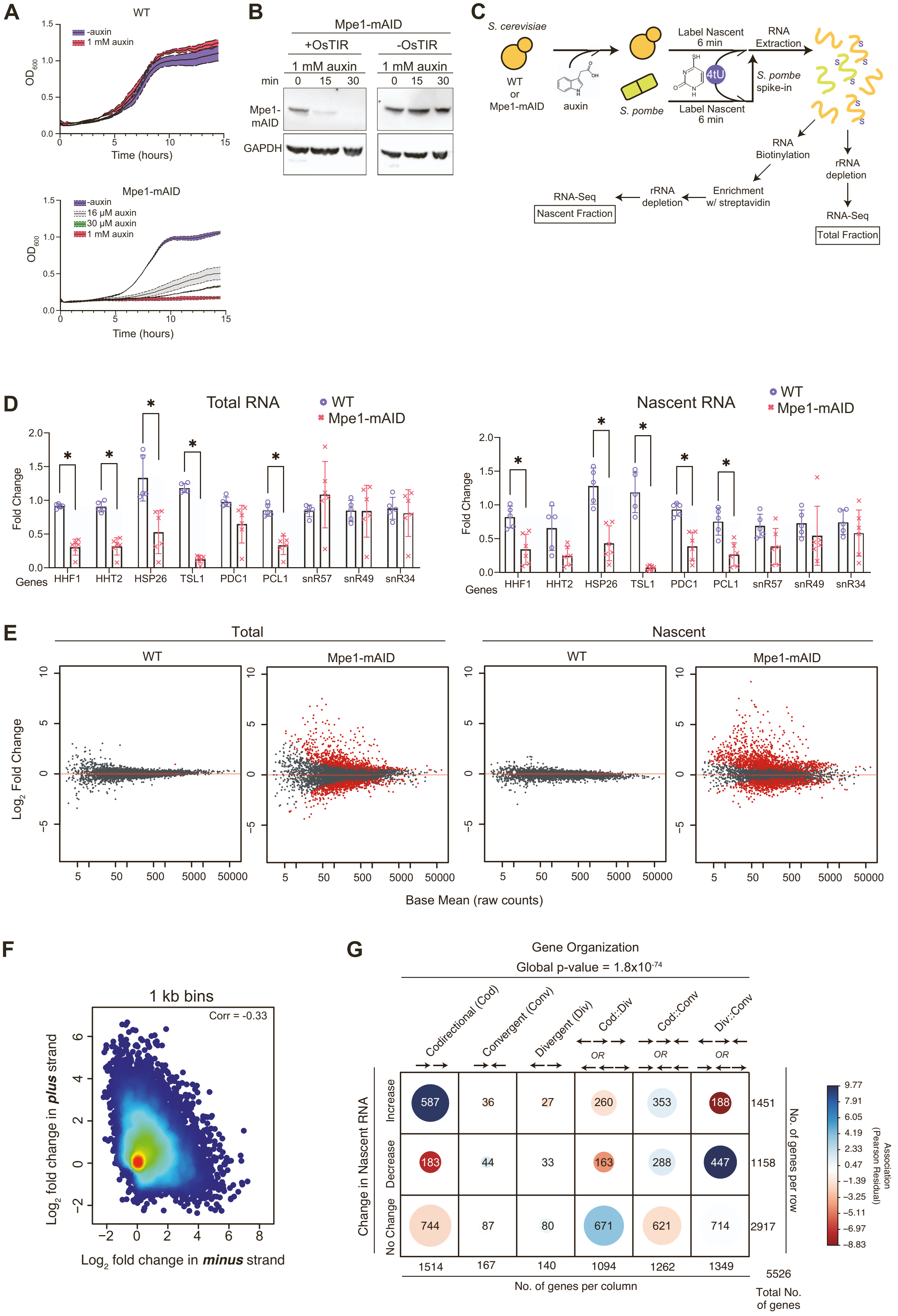
A
WT
-auxin
1 mM auxin
OD600
Time (hours)
Mpe1-mAID
-auxin
16 μM auxin
30 μM auxin
1 mM auxin
B
Mpe1-mAID
+OsTIR
-OsTIR
1 mM auxin
1 mM auxin
min 0 15 30 0 15 30
Mpe1-mAID
GAPDH
C
S. cerevisiae
WT or Mpe1-mAID
auxin
S. pombe
Label Nascent 6 min
Label Nascent 6 min
4tU
RNA Extraction
S. pombe spike-in
RNA Biotinylation
rRNA depletion
Enrichment w/ streptavidin
rRNA depletion
RNA-Seq
Nascent Fraction
RNA-Seq
Total Fraction
D
Total RNA
WT
Mpe1-mAID
Fold Change
Genes HHF1 HHT2 HSP26 TSL1 PDC1 PCL1 snR57 snR49 snR34
Nascent RNA
WT
Mpe1-mAID
Fold Change
Genes HHF1 HHT2 HSP26 TSL1 PDC1 PCL1 snR57 snR49 snR34
E
Total
WT
Mpe1-mAID
Nascent
WT
Mpe1-mAID
Log2 Fold Change
Base Mean (raw counts)
F
1 kb bins
Corr = -0.33
Log2 fold change in plus strand
Log2 fold change in minus strand
G
Gene Organization
Global p-value = 1.8x10-74
Codirectional (Cod)
Convergent (Conv)
Divergent (Div)
Cod::Div
Cod::Conv
Div::Conv
OR
OR
OR
Change in Nascent RNA
Increase
Decrease
No Change
587 36 27 260 353 188 1451
183 44 33 163 288 447 1158
744 87 80 671 621 714 2917
1514 167 140 1094 1262 1349
5526
No. of genes per row
No. of genes per column
Total No. of genes
Association (Pearson Residual)
9.77
7.91
6.05
4.19
2.33
0.47
−1.39
−3.25
−5.11
−6.97
−8.83

Figure S6. Depletion of Mpe1 results in transcription readthrough. Related to Figure 6.
(A) Growth curves of WT or Mpe1-mAID cells grown in the indicated concentrations of auxin. Cultures were grown in three independent biological replicates. Standard deviations for each condition are shown as shaded areas around the mean (solid line in the middle).
(B) Immunoblots of whole cell extracts from cells expressing or lacking the *OsTIR1* gene, treated with 1 mM auxin for the indicated times. Membranes were probed using antibodies against the mAID tag (top panels) or GAPDH as loading control (bottom panels).
(C) Schematic of RNA labeling, and total or nascent RNA library preparation and sequencing. A 4-thiouracil-labeled *S. pombe* spike-in was included as an independent internal control.
(D) Average fold change in selected transcripts from total (left) or nascent (right) RNA fractions upon treatment of WT (purple circles) or Mpe1-mAID (red x) cells with 1 mM auxin for 30 min. N = 5 or 6 as indicated. Error bars represent the standard deviation. *, P-value<0.05 Student's T-test.
(E) MA-plot of transcripts from total or nascent RNA fraction from WT or Mpe1-mAID cells as indicated. Genes that show a significant change in abundance (|$\log_2$ fold change > 0|, FDR-adjusted p-value<0.05) are highlighted in red.
(F) Density scatter plot of the strand-specific $\log_2$ fold change in nascent RNA upon Mpe1 depletion. Points on the scatter plot correspond to the $\log_2$ fold change within a moving 1 kb bin across the genome on each strand.
(G) χ^2 test of independence between changes in nascent RNA upon Mpe1 depletion and gene organization. Genes that increase, decrease or do not change (rows) were classified based on their relationship to their neighboring gene (columns; codirectional (cod), convergent (conv), divergent (div)). Within each row, genes that are shared between two different columns (i.e. cod, conv, div) were classified into hybrid categories (cod::div, cod::conv, div::conv). The number of genes in each classification is illustrated by the size of the circle and the number inside. The color of each circle represents the association between individual row and column categories (Pearson residual; blue or positive values denote positive association, red or negative values denote negative association).

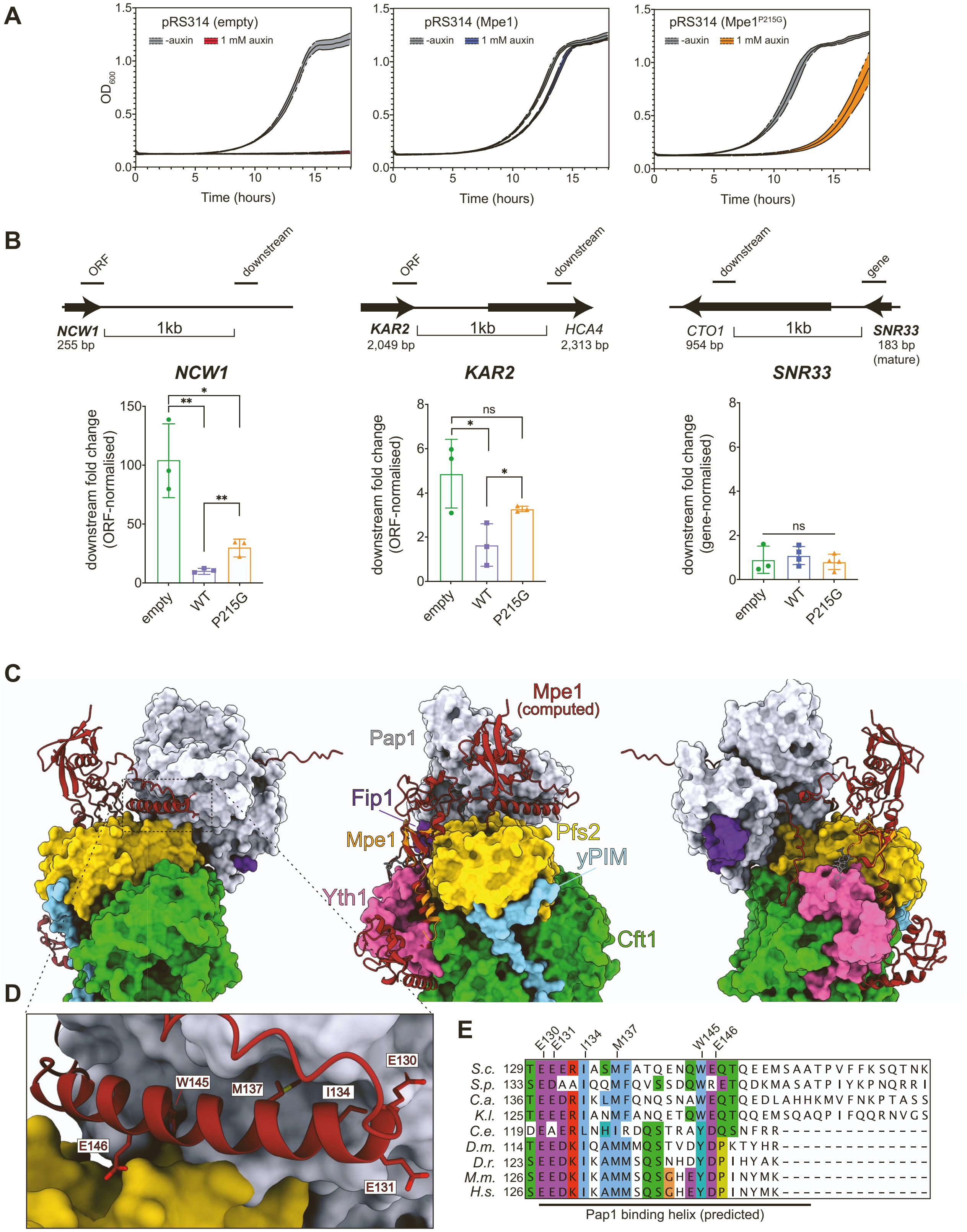

Figure S7. The Mpe1 PSR is important for transcription termination and may help position Pap1. Related to Figure 6 and 7.

(A) Growth curves of Mpe1-mAID cells transformed with the indicated plasmids and treated with 1 mM auxin or an equivalent volume of ethanol (solvent control). For each strain, three independent seed cultures were used

(n = 3 biological replicates). Standard deviations for each condition are shown as shaded areas around the mean (solid line in the middle).

(B) Average fold change in qPCR signal downstream of indicated genes (bold) upon depletion of endogenous Mpe1-mAID and supplementation with WT Mpe1, Mpe1^{P215G} or an empty plasmid control. Two mRNA genes (*NCW1* and *KAR2*) and one snoRNA gene (*SNR33*) were analyzed. Downstream fold changes were normalized to the corresponding fold change within the genic region 1 kb upstream. The total RNA fraction was used for these analyses. Positions for the site of qPCR at the genic region (ORF or gene) and the downstream region are shown as short black lines. Position of genes is shown as black arrows. The length of each gene is shown below each gene name. The value of individual replicates is shown as individual dots. Error bars represent the standard deviation. N = 3-4. *, P-value<0.05, **, P-value<0.01 Student's T-test. ns, no significant difference.

(C) Composite model of the polymerase module in complex with Mpe1 and Pap1. A predicted model of an Mpe1-Pap1 interaction (Humphreys et al, 2021) was first aligned to the polymerase module-Mpe1-yPIM-RNA model by the Mpe1 PSR. A model of Pap1-Fip1 (PDB: 3c66) was then aligned to the Pap1 structure in the predicted Mpe1-Pap1 model. The RING domain of Mpe1 clashes with Yth1 in the model but since the RING is connected by a flexible linker, it could easily move into another position.

(D) The putative Pap1 interacting helix of Mpe1 is shown. Conserved residues within the helix are highlighted.

(E) Multiple sequence alignment of the region of Mpe1 encompassing the putative Pap1 binding helix. Species abbreviations are the same as in Figure 1F and additionally contain sequences of Mpe1 homologues from *Candida albicans* (*C.a.*) and *Kluyveromyces lactis* (*K.l.*). Residues are colored following the ClustalX color scheme (Thompson et al., 1997) implemented in Jalview, which takes into account conserved properties of residues within a given position.

Table S1: Oligonucleotides used in this study. Related to Key Resources Table

**Name**	Sequence
qPCR primers	
HHF1_3_F	CCAAGCGTCACAGAAAGATTC
HHF1_3_R	CGCTTGACACCACCTCTTCT
HHT2_F	TGGTCAGAGAAATCGCTCAA
HHT2_R	GACGGATTCTTGCAAAGCAC
HSP26_F	TGGTTTCGGTTTCCCTAGAA
HSP26_R	GACACCAGGAACCACGACTT
TSL1_3_F	CAGTTGGTATCGACGCCTTT
TSL1_3_R	TTTCCCTTGCCATCTTTCAC
PDC1_+1078_F	CCAGCTTCTACCCCATTGAA
PDC1_+1171_R	AGGTACCGGTTTCAGCAATG
PCL1_F	GCGAACAACACAGTCGCTAA
PCL1_R	CAGATCCCAACTTTGCCTGT
snr57_3_F	TTCTGAGGAAGTATATGCAGGACA
snr57_3_R	CATAAACGAAGAATTCCTAATTCACA
snr49_3_F	TCTCCATGACTATGCCATTTCT
snr49_3_R	TCTACGGGATTCGTTTACCA
SNR34_ +136_F	GGAAGTCCGATTTCTGTGTTG
SNR34_ +189_R	TTCGACATCCCAATTCTATCG
KAR2_ORF_Fwd	CCGCCATTGCTGAAGACTTTGATG
KAR2_3'ORF_Rev	CTACAATTCGTCGTGTTCGAAATAATCACC
KAR2_downstream_Fwd	TGAGGTTTTGACGAAAATAGGTAGTC
KAR2_+1kb_Rev	GCTTGATCTAAATGTTGTAAAATACGACCA
NCW1_ORF_Fwd	TCTACTGCTAGTTCCAGTGCCGCTAAG
NCW1_ORF_Rev	TTAAATACCGGTGCCTAGGACGAAAG
NCW1_downstream_Fwd	TCCATGGGAATAACGATGCACTT
NCW1_downstream_Rev	ACAGAGTAGATTCGTAATAACTTAAATGGC
SNR33_gene_Fwd	TCTAAAATTATTTACGTAATTTAATTGG
SNR33_gene_Rev	GATTGTCCACACACTTCTATATC
SNR33_downstream_Fwd	CTGATAAGGTCAAGATACTGGGTG
SNR33_downstream_Rev	TTAGCAGTACACCATTAGTAGATGG
S_pombe_act1_+111_F	ACCCCGTCACCATGGTATTA
S_pombe_act1_+186_R	ACGCTTGCTTTGAGCTTCAT
S_pombe_adh1_+331_F	GAGACCATCTGCCCTCACAT
S_pombe_adh1_+402_R	GGCAATGCAGTAGTGTTGGA
S_pombe_gpd3_+892_F	TCTGCCGGTATCCAACTTTC
S_pombe_gpd3_+977_R	TCAACAACACGGTGGGAGTA
Primers used for strain construction	
Mpe1_F2*	ACG GCT ACT ATC ACA AAT CCT CAT CAA GCT GAC GCA AGC CCT AAG AAA CGG ATC CCC GGG TTA ATT AA
Mpe1_R1*	ACGTATGTGAAGCCAAGTAGGCAATTATTTAGTACTGTCAGTATT GTTAT GAATTCGAGCTCGTTTAAAC
Mpe1_tag_check_F**	CGCTGGCTTGAACAACAATA
KanB_deletion_check**	CTGCAGCGAGGAGCCGTAAT
OsTIR1_check_F^	GGGATGCAAGTCCAGTTTGT
OsTIR1_check_R^	ACGATTGCACCAAAACCTTC
Oligos used for in vitro transcription	
R00_T7_Fwd	TAATACGACTCACTATAGGGTTTATAGTTATGTTAGTATTAAGAAC GTTATTTATATTTCAA
R00_T7_Rev	TTGAAATATAAATAACGTTC**T**TAATACTAACATAACTATAAACCCT

	ATAGTGAGTCGTATTA
Primers used for bacmid vector construction	
pIDS_CasI_F	AACGCTCTATGGTCTAAAGATTTAAATGGCACCTAGGTATCGATACTAGTATAC
pIDS_CasI_R	AAACGTGCAATAGTATCCAGTTTATTTAAATGTACCCGTAGTGGCTATGGCAGGG
pIDS_CasII_F	AAACTGGATACTATTGCACGTTTAAATGGCACCTAGGTATCGATACTAGTATAC
pIDS_Casω-R	AACCCCGATTGAGATATAGATTTATTTAAATGTACCCGTAGTGGCTATGGCAGGG
pB/pIDC_CasI_F	AACGCTCTATGGTCTAAAGATTTAAATCGACCTACTCCGGAATATTAATAGATC
pB/pIDC_CasI_R	AAACGTGCAATAGTATCCAGTTTATTTAAATGGTTATGATAGTTATTGCTCAGCG
Mpe1_F9A_pIDS_F	AGTCAACATGAGCAGCACCATCTTTTATCGT**GCC**AAAAGCCAGCGTAATACCAGCCGCATTCT
Mpe1_F9A_pIDS_R	AGAATGCGGCTGGTATTACGCTGGCTTTT**GGC**ACGATAAAAGATGGTGCTGCTCATGTTGACT
Mpe1_R76E_P78G_pIDS_F	CGCTAAAGCTTTTAATTGC**CCC**ACT**CTC**TTTAACAATAACGCTGGTGCTACGCGGAATAA
Mpe1_R76E_P78G_pIDS_R	TTATTCCGCGTAGCACCAGCGTTATTGTTAAA**GAG**AGT**GGG**GCAATTAAAAGCTTTAGCG
Mpe1_D45K_pIDS_F	GAACAAACTGGGTGATGGCACC**AAG**TTCCAGCTGAAAATCTATAATC
Mpe1_D45K_pIDS_R	GATTATAGATTTTCAGCTGGAA**CTT**GGTGCCATCACCCAGTTTGTTC
Mpe1_W257A_Y260A_F	GTCCAGGTGGAAGATAAACAGAGC**GCA**GAAGAT**GCA**CAGCGTAAACGTGAAAATCGTCAG
Mpe1_W257A_Y260A_R	CTGACGATTTTCACGTTTACGCTG**TGC**ATCTTC**TGC**GCTCTGTTTATCTTCCACCTGGAC
Mpe1_P215G_F	CGTATTCGTCGTACCACCGGCATT**GGT**AAAAAATTTCTGAAAAGCATCGAG
Mpe1_P215_R	CTCGATGCTTTTCAGAAATTTTTT**ACC**AATGCCGGTGGTACGACGAATACG
pACE_Mpe1_Zn_Fwd[@]	CCGTCCCACCATCGGGCGCGGATCCATGTTCAAAAGCCAGACCAATAAAAACAGCGC
pACE_SII_Mpe1_Zn_Rev[#]	CCTGGAAATACAGGTTTTCCTCGAGCTGACGATTTTCACGTTTACGC
ZnKdelta_Fwd[@]	ACTCCGGTTTTTTTCAGCGATCCGAACTTTGAAGG
ZnKdelta_Rev[#]	AAAGTTCGGATCGCTGAAAAAAACCGGAGTTGCTG
PSRdelta_Fwd[@]	GAAGGTAAACGTATTCGTCAGATTGATGGTGATGA
PSRdelta_Rev[#]	ACCATCAATCTGACGAATACGTTTACCTTCAAAGT
paceCft2_fwd	CCGTCCCACCATCGGGCGCGGATCCATGACCTATAAATACAATTGTTGTG
paceCft2_rev_no_stop_codon	CCTGGAAATACAGGTTTTCCTCGAGAATTTTGGCCAGCATATCTG
Cft2_F537A_Y549A_F558A_Fwd	CAGCCGAGCGCAGCAAGCAAACATAAAATGGCCCCGTTTAACCCTGCCAAAATCAAAAAAGATGATGCCGGCACCGTGGTGGACTTTACCATGGCCCTGCCGGATGATAGCGATAATGTTAATCAG
Cft2_F537A_Y549A_F558A_Rev	CTGATTAACATTATCGCTATCATCCGGCAGGGCCATGGTAAAGTCCACCACGGTGCCGGCATCATCTTTTTTGATTTTGGCAGGGTTAAACGGGGCCATTTTATGTTTGCTTGCTGCGCTCGGCTG
pACE_Mpe1_F[@]	CCGTCCCACCATCGGGCGCGGATCCATGAGCAGCACCATCTTTTATC
pACE_SII_Mpe1_R[#]	CCTGGAAATACAGGTTTTCCTCGAGTTTTTTCGGGCTTGCATC

pACE_Mpe1_R[@]	TTCCGCGCGCTTCGGACCGGGATCCTTATTTTTTCGGGCTTGC

*Underlined sequences anneal to the pST1933 vector
**Primers used for screening potential Mpe1-mAID colonies
^Primers used to test presence of *OsTIR1* gene
Nucleotides in bold insert the indicated mutation
[@]Underlined sequences anneal to the 5' (F) or 3' (R) end of the Mpe1 coding sequence.
[@]Primers used for subcloning Mpe1 and Mpe1 into pACEBAC1 linearized with BamHI.
[#]Used for subcloning Mpe1 and Mpe1 variants into pACEBAC1-TEV-SII linearized with BamHI/XhoI.
